# A framework to automatically detect near-falls using a wearable inertial measurement cluster

**DOI:** 10.1038/s44172-024-00325-x

**Published:** 2024-12-16

**Authors:** Maximilian Gießler, Julian Werth, Bernd Waltersberger, Kiros Karamanidis

**Affiliations:** 1 Department of Mechanical and Process Engineering, Offenburg University of Applied Sciences, Offenburg, Germany; 2 Sport and Exercise Science Research Centre, School of Applied Sciences, London South Bank University, London, UK; 3 Department of Sport Science, Faculty of Mathematics and Natural Sciences, University of Koblenz, Koblenz, Germany

**Keywords:** Public health, Biomedical engineering, Electrical and electronic engineering, Mechanical engineering, Computational science

## Abstract

Accurate and automatic assessments of body segment kinematics via wearable sensors are essential to provide new insights into the complex interactions between active lifestyle and fall risk in various populations. To remotely assess near-falls due to balance disturbances in daily life, current approaches primarily rely on biased questionnaires, while contemporary data-driven research focuses on preliminary fall-related scenarios. Here, we worked on an automated framework based on accurate trunk kinematics, enabling the detection of near-fall scenarios during locomotion. Using a wearable inertial measurement cluster in conjunction with evaluation algorithms focusing on trunk angular acceleration, the proposed sensor-framework approach revealed accurate distinguishment of balance disturbances related to trips and slips, thereby minimising false detections during activities of daily living. An important factor contributing to the framework’s high sensitivity and specificity for automatic detection of near-falls was the consideration of the individual’s gait characteristics. Therefore, the sensor-framework presents an opportunity to substantially impact remote fall risk assessment in healthy and pathological conditions outside the laboratory.

## Abstract (web summary)

Maximilian Gießler and colleagues present a framework for detecting and distinguishing near-falls related to trips and slips using a wearable sensor. Their system accounts for individual gait characteristics, thereby minimising false detection.

## Introduction

Fall injuries are recognised as a major public health concern affecting all population groups, but particularly older adults. Injuries not only result in physical harm but also have far-reaching social and financial implications^1,2^. Given approximately every 10th adult over 40 years may fall at least once a year^3,4^, with the incidence reported majorly during locomotion (e.g. caused by trips, slips or stumbling^5,6^, precise monitoring of kinematic patterns during daily movement becomes crucial for both developing prevention strategies, e.g. advanced training methods, and assessing their effectiveness.

To monitor the kinematics of human body segments during daily life locomotion, wearable sensors incorporating gyroscopes and/or linear accelerometers (so-called inertial measurement unit (IMU)) are commonly used^7,8^. A major advantage of using wearable sensors for monitoring balance disturbances (e.g. trips or slips) that often lead to falls or near-falls during activities of daily living (ADLs), as opposed to current applied approaches such as questionnaires, is the objective data-driven analysis. Questionnaire-based approaches are biased likely due to subjective or inaccurate reporting^9^. Wearable sensors combined with evaluation algorithms, in turn, have been extensively examined with the aim of achieving objective and automatic detection of falls and distinguishment of fall events from ADLs, respectively^10–13^. These approaches typically utilise sensors attached to various anatomical landmarks to monitor body kinematics, such as a bi-axial gyroscope or three-axis accelerometer mounted at the trunk or chest^10–12^, or an IMU combination attached to the thigh and helmet^13^. Furthermore, recent advancements in machine learning and deep learning have shown promising results, particularly in the field of automated fall detection, with improvements in the accuracy of wearable sensor-based detection systems^13–15^. For example, Liu et al.^14^ as well as Gomaa and Khamis^15^ reviewed the potential of both classical machine-learning models and deep-learning architectures to enhance the accuracy and reliability of fall detection.

However, while the automatic detection of falls and the distinguishment of fall events from ADLs have been addressed widely, there is limited information on the use of wearable sensors to accurately assess near-fall scenarios in daily life settings, e.g. caused by tripping or slipping; a feature which would increase the sensitivity of early diagnosis of balance and gait dysfunctions in various populations. Wearable sensor-based and machine-learning approaches for near-fall detection do exist, with studies showing that both classical machine-learning algorithms and deep-learning models can effectively classify near-fall events and offer a promising approach to fall prevention^16,17^. However, there is a lack of information on their application outside controlled laboratory environments, particularly in distinguishing near-falls from ADLs. These approaches may be limited by the need for comprehensive labelled training data, encompassing various types of falls, near-falls and ADLs, to ensure accurate classification during remote monitoring. Moreover, most current approaches to automatic fall or near-fall detection via wearable sensors neglect inaccuracies introduced by inter-subject variability in movement patterns, as well as intra-subject changes in locomotion kinematics, such as those occurring post-injury or due to pathology^10–12^.

A further limitation of current wearable sensors-based approaches refers to the requirement of a noise-amplifying numerical differentiation of the measured angular velocity to determine the angular acceleration vector^18^. In addition, the regulation of trunk dynamics and whole-body angular momentum is an essential component of withstanding forward-directed fall initiations^19–21^. Given the trunk’s anatomy, its motion patterns are dominated by rotational kinematics. To accurately quantify and resolve the trunk dynamics, knowledge of the angular acceleration vector is key. However, the wearable sensor-based approaches lack direct measurements of the angular acceleration vector, potentially leading to an ineffective detection of balance recovery responses related to near-fall scenarios. Ultimately, approaches for distinguishing between a wide range of perturbation magnitudes during locomotion that lead to balance loss (i.e. near-falls) and ADLs have not yet been reported. Thus, the development of a sensor-framework which is capable of both detecting trip- and slip-like events (near-falls) and distinguishing these from ADLs would advance those approaches solely providing the ability to distinguish between ADLs and falls.

Recently, we introduced a framework and an inertial measurement cluster (IMC) that do not require numerical differentiation to directly assess trunk angular acceleration vectors during perturbed locomotion^18^. The wearable IMC combined with a framework allows for an automatic and remote assessment of anterior fall initiation and balance recovery adaptations to repeated perturbations^18^. The objective of the current study was to expand our recent approach and progress on the IMC. We aimed to demonstrate its feasibility for both precise and automatic distinguishment between perturbed locomotion patterns induced by various types of external perturbations (e.g. trips and slips) and ADLs (e.g. walking and running, staircase locomotion, sit-to-stand and stand-to-sit, pick-and-drop), as well as their classification in terms of trips, slips and antero-posterior loss of balance. Although there is no universally agreed-upon definition for near-falls, they are generally considered as a loss of balance that is recovered before a fall occurs. Recovery involves a neuromuscular reaction that successfully prevents a fall. We propose defining near-falls by categorising anomalies in trunk kinematics within specific boundaries. The thresholds for the upper boundary distinguish a near-fall from a fall. Thereby, a fall is defined as an unintentional and forceful contact with the ground, excluding the feet. The lower boundary is personalised to consider individual’s gait characteristics, defined by thresholds in magnitude and frequency content of anomalies in trunk kinematics.

An important issue of the current work was to examine the potential impact of higher accuracy in kinematics extractions via the IMC on the automatic detection and classification of trip- and slip-related balance perturbations. Therefore, we additionally used and compared the outcomes of a single IMU as opposed to the IMC using our framework. Based on a direct hence more accurate assessment of the trunk angular acceleration vector during locomotion (no numerical differentiation required), we hypothesised that the IMC would enhance accuracy in near-fall detection, reducing false detections of trips and slips amongst ADLs.

## Results

### Sensitivity and specificity to assess balance perturbations using the IMC

**Fig. 1:**
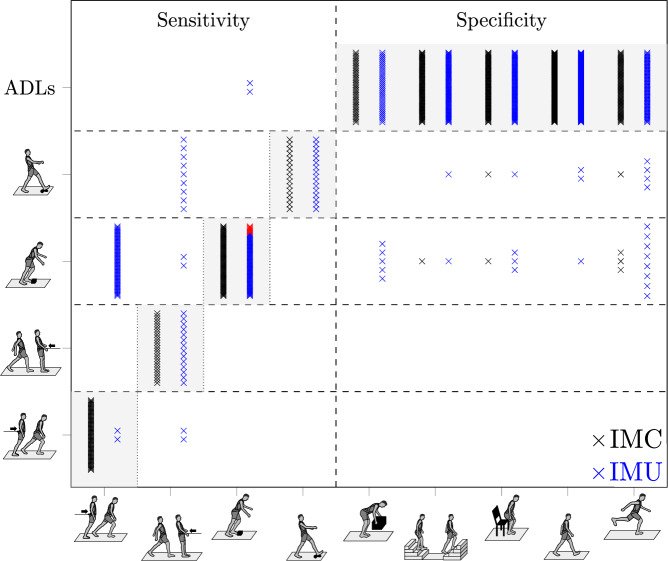
Matrix scheme of the automated distinguishment and near-fall type classifications during locomotion by the framework. The results of the framework in combination with the inertial measurement cluster (IMC) measurements for each of the 627 trials (*y*-axis) are represented by black crosses and plotted against the corresponding original exercise (*x*-axis). The framework’s results using a single inertial measurement unit (IMU) measurements for each of the 627 trials (*y*-axis) are represented by blue crosses. Red crosses indicate that more perturbed locomotion events were identified than were actually applied in the respective IMU measurement trials. The corresponding classification of the framework is shown on the *y*-axis ordered in anterior loss of balance; posterior loss of balance; trip; slip; ADLs (from bottom to top). ADLs refer to the summary of all simulated daily activities. The corresponding original exercise (*x*-axis) is indicated by stick figures (from left to right): anterior loss of balance; posterior loss of balance; trip; slip; pick-and-drop; staircase walking; sit-to-stand; walking; running. The main diagonal of the matrix (shaded in grey) represents correct classifications.

**Fig. 2:**
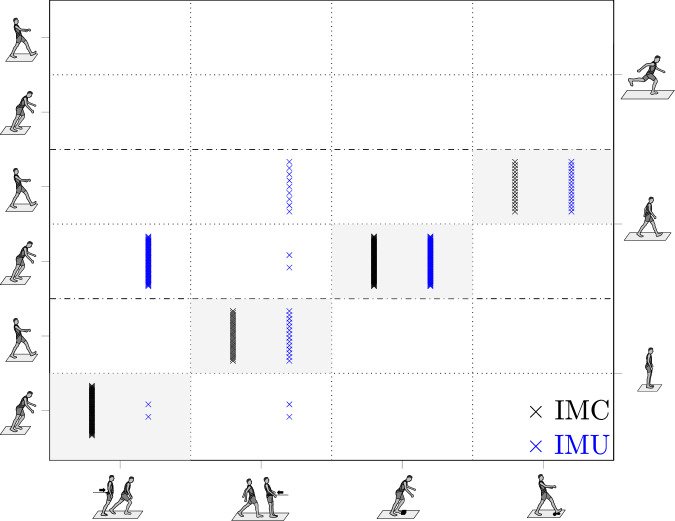
Matrix scheme of the automated classification of detected perturbations during locomotion by the framework. The results are divided into the classifications relying on the inertial measurement cluster (IMC; black crosses) and inertial measurement unit (IMU; blue crosses) kinematics. The applied types of perturbations are presented on the bottom axis with stick figures (from left to right): anterior loss of balance; posterior loss of balance; trip; slip. The *y*-axis (left) indicates the classification results, i.e. whether the initial trunk movement was directed anteriorly or posteriorly. The *y*-axis (right) specifies the identified preceding locomotion with respect to the perturbation onset. The main diagonal of the matrix (shaded in grey) represents correct classifications.

A total of 627 human locomotion trials were analysed, including 179 perturbed trials. The perturbed trials consisted of 74 trip- and 16 slip-like perturbations during walking, 62 and 27 trials of balance loss in antero-posterior directions from a forward or backwards leaning task during quiet bipedal stance, respectively. Out of the remaining 448 ADLs trials, 84 consisted of sit-to-stand and stand-to-sit, both followed by preferred walking; 168 level walking and 72 running trials; 82 ascending and descending staircase trials; and 42 pick-up and drop walking trials. The results regarding the sensitivity and specificity of the IMC and single IMU are summarised in Supplementary Table S1. Additionally, the acquired number of perturbations and ADLs including the true negative detections per participant were summarised in Supplementary Tables S3 and S4. With respect to the sensitivity analysis for the IMC, the algorithm was capable to correctly detect all 179 antero-posterior perturbation trials during standing and walking, i.e. 100% sensitivity (Fig. 1). Accordingly, a variation of balance slip- and trip-perturbation magnitudes induced by changes in gait velocity and antero-posterior inclination angles (for a lean-and-release task) did not affect sensitivity. Out of all ADLs trials (*n* = 448), the algorithm revealed a total of seven false-positive detections, i.e. specificity of 98.4% using the IMC (Fig. 1). For these false-positive detections, one was present in ascending stairs, two in sit-to-stand and stand-to-sit and four in running. With the IMC resulting in 179 true-positive and seven false-positive detections, the positive predictive value (conditional probability) of actual perturbed locomotion was 96.2%. The F1-score resulted in 98.1%, and the prevalence for perturbed trials in the entire set was 28.5% (cf. section ‘Statistics’). Concerning the classification of trips, slips or antero-posterior loss of balance during stance, as well as the type of movement (standing, walking or running) immediately preceding to the onset of the perturbation, our framework revealed 100% of correct predictions (Fig. 2). Note that for trials including running, no external perturbation was triggered.

### Sensitivity and specificity to assess balance perturbations using a single IMU

To determine the potential impact of a single IMU on the assessment of balance recovery response, we additionally conducted an identical test procedure for all measured locomotor trials using kinematic measurements of one single IMU Xsens MTi-20 VRU (Movella Inc., Nevada, USA). We evaluated the angular acceleration curve path using both the central difference quotient and the difference quotient with the same framework. Accordingly, the personalised threshold values of trunk angular acceleration were extracted using numerically differentiated signals of the baseline curves. Using the central difference quotient, the framework revealed two misdetections out of the 179 perturbation trials for trips and slips during walking, and balance loss during stance using a single IMU, i.e. sensitivity of 98.9% (Fig. 1). With regards to the 74 trip- and 16 slip-like perturbations trials during walking, the misdetections were characterised by two false-negative and eight false-positive evaluations. In cases of false positives, the framework identified more perturbed locomotion events than were actually applied in the respective trials (indicated by red crosses). However, given 98.9% sensitivity for the wide range of analysed balance perturbations during standing and walking, the results show that the sensitivity of the IMU was not affected by the perturbation magnitude. Regarding the 448 ADLs trials, the framework showed a total of 34 false-positive detections, i.e. a specificity of 92.4% using a single IMU (Fig. 1). Out of these false-positive detections trials, five were present in pick-up and drop walking, two in ascending and descending stairs, four in sit-to-stand and stand-to-sit, one in preferred and two in fast walking and 12 in running. The positive predictive value of actual perturbed locomotion was 83.9%, with 177 true-positive and 34 false-positive detections. The F1-score resulted in 90.8%. The distinguishment of the type of perturbation (trip, slip, anterior or posterior loss of balance) showed 58.1% of correct predictions. For the distinguishment between standing, walking or running, the motion pattern immediately preceding the onset of the perturbation was correctly classified in 59.2% out of all 179 perturbed trials (Fig. 2). In 73 trials of losing balance during stance, the type of movement immediately preceding the onset of the perturbation was identified as walking. This resulted in incorrect evaluations, i.e. the framework detected trips or slips instead of loss of balance during stance. When using the backward difference quotient to extract the angular acceleration signal, the framework revealed a total of 13 misdetections in perturbed walking trials, with five false-negative and eight false-positive evaluations, i.e. 97.2% sensitivity. The specificity resulted in about 89.7%, with a total of 46 false-positive detections. Out of these false-positive detection trials, eight were present in pick-up and drop walking, two in ascending and descending stairs, three in sit-to-stand and stand-to-sit, one in preferred, three in fast walking and 20 in running. Regarding all 179 perturbed trials, the type of perturbation and the distinguishment between standing, walking or running resulted in 52.2% of correct predictions. In 85 trials of losing balance during stance, the type of movement immediately preceding the onset of the perturbation was detected as walking.

### Effects of IMC and IMU on personalised thresholds

To further examine the differences in sensitivity and specificity between both approaches (IMC vs. single IMU), we analysed the personalised thresholds. We compared these thresholds for the averaged local and global maximum of angular acceleration signal assessed by IMC and Xsens MTi-20 VRU, using the same threshold determination algorithms. Using the central difference quotient and backward difference quotient, respectively, the global maximum threshold retrieved from the IMU showed an averaged deviation factor of 1.1 and 1.3 compared to the IMC. Thresholds of individuals revealed a maximum deviation of 1.76 or 2.11 and a minimum of 0.66 or 0.7, respectively. The local maximum showed an averaged deviation factor of 0.98 or 1.1, with the individual’s maximum and minimum deviation of ~1.3 or 1.4 and 0.76 or 0.8, respectively.

## Discussion

An accurate extraction of body segment kinematics via wearable sensors is essential to provide new insights into the complex interactions between active lifestyle and fall risk in various population groups. However, an inherent limitation of current approaches to assess the angular acceleration vector of various body segments is the noise-amplifying numerical time derivative required^22,23^. Although wearable sensor-based machine-learning approaches for near-fall detection exist and have shown promising results in effectively classifying near-fall events^16,17^, there is a lack of information on their capability, particularly in distinguishing near-falls from ADLs in daily life. Ultimately, it is not yet well established as to whether an objective data evaluation via kinematics may be sufficient in sensitivity and specificity to replace current subjective approaches such as questionnaires for the assessment of gait perturbations and near-falls related to trips and slips in daily life. In a recent work, we developed a framework which is sensitive enough to identify well-known gait adaptational phenomena to repeated trip-like perturbations^18^. With the current study, we aimed to expand our research and increase accuracy in joint kinematic analyses for the automatic detection of trips, slips and other postural perturbations within a sequence of various ADLs. We were able to show that our IMC-based approach had a 100% sensitivity and only seven false positives out of 448 ADLs, providing 98.4% specificity. Furthermore, the IMC correctly classified the types of perturbations (slipping, tripping and antero-posterior stability loss from quiet standing) across all evaluated locomotion trials. Moreover, our IMC-based approach was also able to detect moderate-induced trips, slips and balance perturbations during stance demonstrating a robust assessment. These findings prove that the proposed framework provides an accurate assessment of trips or slips, and hence may help to objectively evaluate daily life fall risk in various populations. However, due to the small number of participants (18 participants), we were unable to draw direct conclusions about the framework’s accuracy in detecting near-falls across different population groups, including variations in age, physical condition and health status.

A reasonable factor contributing to the high sensitivity and specificity of our framework was the consideration of the individual’s gait characteristics via personalised thresholds. Even though the examined subject group was quite homogeneous with respect to anthropometric characteristics, age, physical fitness and health status, the sample revealed fairly wide ranges of both global maximum and averaged local maximum in trunk kinematics during unperturbed walking at a given speed (28–84 rad/s^2^ and 19–46 rad/s^2^, respectively), indicating that the integration of personalised thresholds is crucial for trip and slip detection. Furthermore, it is important to note that we did not generically classify potential trip- or slip-like events by the trunk kinematics’ curve intervals by simply considering exceeded personalised threshold levels (i.e. necessary condition). Instead, we conducted further automatic subsequent data analysis of the observed curve interval related to any exception to identify additional predefined criteria (i.e. sufficient condition) in the trunk kinematic characteristics (see ‘Methods’ section ‘Subsequent data analysis to distinguish between perturbed motion and ADLs’). For example, an ADLs task such as running would have been misclassified as a trip if personalised threshold levels in trunk kinematics were exceeded by factor 4 (e.g. the averaged global maximum threshold for our examined subjects was 181 rad/s^2^ and 44 rad/s^2^ for running and unperturbed baseline walking, respectively). Thus, we state that an accurate distinguishment between trips, slips or other balance perturbations and ADLs requires consideration of both, personalised thresholds, and subsequent data analysis of the observed curve interval.

When using the current framework, one might argue that a state-of-the-art wearable sensor incorporating a single IMU with a central difference quotient is sufficient to automatically assess and distinguish between trips or slips and ADLs, given the IMC-based approach showed only marginal advantages in sensitivity (100% vs. 98.9%) or specificity (98.4% vs. 92.4%). However, the evaluated positive prediction (conditional probability) for balance recovery response was clearly lower using an IMU (83.3%) compared to our IMC (96.2%), even for the central difference quotient (study prevalence rate of 28.5%). Such differences in the positive prediction value of the approaches must be considered as particularly relevant for the detection of trips and slips during daily life when the prevalence of such events is low. To date, there is no adequate information available which refers to the frequency of near-fall incidence during daily life in various populations. Nevertheless, if we provided a fictive prevalence rate of 5%, an extrapolation of the positive detection value would result in a factor-two improvement when using the IMC (IMC 76.7% vs. IMU 40.7%, i.e. four vs. eight out of ten correct detections), with even greater relative differences considering the decrease in near-fall prevalence rate. More importantly, the IMU-based approach showed clear limitations in an accurate distinguishment between trip- and slip-based perturbations and other balance perturbations during stance (73 out of 89 vs. zero misdetections via IMU vs. IMC), despite using the same framework. Thus, next to personalised threshold and subsequent data analysis, an additional key feature of achieving such high accuracy metrics by the IMC combined with our framework was the joint analysis of the trunk angular velocity and directly measured angular acceleration.

The limitations in the metrics and in classifications of an IMU-based approach can be attributed to the negative effects of the required numeric. Our data indicate that the differences in sensitivity or specificity between our IMC and IMUs primarily stem from the use of noise-amplified trunk angular acceleration signals in the latter. Amplified noise resulted in averaged thresholds of 1.1 to 1.3 times higher when using either the central difference or backward difference quotient compared to the IMC’s evaluated thresholds. This led to a reduced bandwidth of detectable balance recovery responses hence false-negative detections for low perturbation magnitudes. Moreover, it must be emphasised that both difference quotient methods act as filters. The IMU approach based on the central difference quotient yielded enhanced sensitivity and specificity, but trials of false-negative detection varied due to different numerical derivative methods, highlighting the potentially negative impact of the numerical derivative methods on accuracy, and the risk of ignoring relevant motion behaviour characteristics (Supplementary Fig. S1). On a similar note, a lower specificity and substantial number of misdetections for trips and slips in locomotor tasks also relied on amplified noise along the IMU-based approach. Due to the stochastic characteristic of sensor signal noise, unfavourable noise term combinations in adjacent angular velocity signal sample points can occur and in fact induce artefacts like local extrema, also mimicking an outlier characteristic when subjected to numerical differentiation. If these artefacts occur simultaneous to daily life activities characterised by increased upper body angular velocity amplitudes (such as pick-up and drop as well as running), it simultaneously implies a necessary and sufficient condition for balance recovery responses. Thus, it causes false-positive detections; in fact, currently present in five trials with central difference quotient as well as eight with backward difference quotient for pick-and-drop, and in 12 trials with central difference quotient as well as 20 with backward difference quotient for running.

To accurately classify trips and slips among other balance perturbations, the proposed automated analysis also required an identification of the preceding locomotion with respect to the perturbation’s onset. For quiet standing trials, our data clearly showed that the trunk’s angular acceleration had low magnitudes, leading to a low signal-to-noise ratio in the numerically derived signal. The induced artefacts mimicking local extrema led to incorrect predictions of the type of perturbation, i.e. amplified noise would be misinterpreted as touchdown-induced local maxima of a walking pattern. This is because local extrema artefacts induced by numerical differentiation showed magnitudes comparable to local maxima, such as those caused by the foot’s touchdown during slow walking. Furthermore, we understood that the analysis limited to the transverse axis’ angular velocity signal fails to identify trips and slips among other balance perturbations. This was caused by the similarity of present local extrema magnitudes through moderate trunk movements in quiet bipedal stance or slow and preferred walking (Supplementary Fig. S2). However, local extrema in the directly measured angular acceleration signal (IMC) provided a more distinct differentiation criterion (Supplementary Fig. S2). This supports our hypothesis that accurate angular acceleration measurements are essential to distinguish between ADLs and near-falls, and to accurately classify trips and slips among other balance perturbations. Although the sensor-framework primarily focused on detecting and distinguishing trips and slips, its sensitivity in detecting general loss of balance, e.g. from unstable body configurations during quiet stance, suggests its potential applicability to a broader range of locomotor activities. A key aspect was the definition of a near-fall, which was based on thresholds for magnitude and frequency content of anomalies in trunk kinematics that were not specifically tied to walking as a locomotor task.

Regarding the measured kinematics (i.e. the underlying sensing principle), the primary objective was to develop an effective system by utilising a minimum number of inertial sensor devices and kinematic signals for remote trip and slip detection. Therefore, the framework was based on the angular velocity and acceleration vectors (rotational kinematics) from the IMC. Unlike linear acceleration, angular kinematics are less sensitive to different sensor locations and therefore more suitable for long-term monitoring. Moreover, the framework relies solely on rotational kinematics, as the trunk’s movement is primarily dominated by rotational dynamics due to musculoskeletal anatomy. The outcomes of the IMC-based evaluation demonstrated that exploiting the angular velocity and the directly measured angular acceleration vectors was sufficient to detect and distinguish between near-falls and ADLs, and to accurately classify trips and slips among other balance perturbations. This approach enabled the use of a minimal set of rotational kinematic data, thereby simplifying the framework.

Finally, it is important to note that previous research on inertial sensors has primarily focused on fall detection and distinguishing falls from ADLs, rather than on the assessment of near-falls due to tripping or slipping in daily life. Using parameter extractions from accelerometer and gyroscope readings, several studies^10–13,24,25^ showed that threshold-based algorithms or machine-learning techniques have the potential to identify falls with relatively high sensitivity (>90%) and specificity, respectively. We, in turn, provided evidence that metrics within our framework (i.e. sensitivity, specificity or positive predictive value) aiming to identify situational balance loss are as accurate as fall detections. Further, in identifying also moderate-induced perturbations, we considered a wide range of perturbation magnitudes during locomotion, and thereby confirmed the effectiveness of the framework. Thus, we postulate that our framework provides accurate measures and may pioneer daily life falls risk assessment in clinical and research settings.

Considering the limitations of this study, the efficacy of the proposed approach was validated using standardised antero-posterior trip- and slip-like perturbations, along with a broad range of simulated ADLs in a controlled setting. However, daily life involves a wider spectrum of movement patterns, with perturbations occurring in arbitrary spatial directions, such as mediolateral perturbations. Therefore, it is essential to evaluate the accuracy metrics and applicability under less controlled conditions of the proposed approach. Nevertheless, the algebraic and mechanically interpretable structure of the framework will aid in handling a broader range of perturbations and motion patterns expected during daily life.

Next to this, the approach’s validation was conducted with a homogeneous participant group. The findings and applicability must be further validated with a more heterogeneous group in terms of age, physical condition and health status. In fact, the framework will benefit from consideration of the participants’ specific gait characteristics via personalised thresholds. A further potential refinement is the calculation routine for personalised thresholds. Currently, these thresholds are globally calculated once based on the baseline curves. For long-term measurements in varying environmental conditions, an adaptive adjustment routine could be incorporated to update the personalised thresholds during the measurement period. This adjustment could be triggered manually by the participant to account for noticeable changes in environmental conditions, physical condition or health status. However, the proposed definition of a near-fall—based on trunk kinematic anomalies and guided by necessary and sufficient conditions—will be crucial in preventing false-positive detections, especially during long-term monitoring in daily life settings, where the individuals’ gait characteristics may vary. In particular, the use of necessary and sufficient conditions helps to reduce the framework’s reliance on personalised thresholds derived from baseline measures, as these personalised thresholds are primarily relevant for identifying necessary conditions. In addition, its algebraic structure allows for further personalisation, such as individual-specific adaptation of the structure or the subroutines, which could improve the handling of inter-subject differences in gait behaviour.

Another limitation is the current experimental prototype’s size and weight, which may restrict the individual’s mobility. Future work will aim to miniaturise the IMU system, despite the challenges posed by stochastic noise amplification (Eq. 1) and the deterministic errors due to mispositioning^18^. To mitigate these issues of miniaturisation (increasing measurement uncertainty), we will address the deterministic errors compensation by using a mechanical angular acceleration reference to calibrate and adjust parametric error models of the IMC, thereby reducing measurement uncertainty. Moreover, future research could enhance the framework by incorporating additional wearable sensors (e.g. single IMUs for linear acceleration measurements) on other body segments, such as the feet or shank, to further improve the distinguishment between balance recovery responses and ADLs.

## Conclusion

This study indicates that the proposed wearable IMC and framework combination for analysing trunk kinematics can be used to automatically identify antero-posterior stability disturbances and near-falls during daily life tasks. The key advantage of the IMC over IMU solutions is the accurate identification of trips and slips during walking. Thereby, the direct measurement of trunk angular acceleration, the use of personalised thresholds and the subsequent analysis of the data observation windows were fundamental for accurate distinguishment between balance perturbations and ADLs, and to avoid false detections particularly those referring to trips and slips. The proposed approach may therefore be used during long-term monitoring in healthy and pathological conditions to accurately assess trips, slips and near-falls outside the laboratory.

## Methods

### Participants and experimental design

Eighteen healthy and moderately physical active young adults participated in this study (data as averages and standard deviations for eight females and ten males; ♀ 26 ± 4 and ♂ 31 ± 8 years of age, ♀ 1.65 ± 0.06 m and ♂ 1.79 ± 0.10 m height, ♀ 66.3 ± 7.7 kg and ♂ 80.6 ± 10.4 kg body mass, ♀ 24.4 ± 2.8 and ♂ 25.2 ± 3.0 body mass index). Potential participants were excluded if they had any neurological or musculoskeletal injuries or impairments to avoid bias caused by problems related to locomotion, whether from disease, or trauma. The study was reviewed and approved by the ethics committee of the School of Applied Sciences at London South Bank University (approval ID: SAS1826b) and met all requirements for human experimentation in accordance with the Declaration of Helsinki^26^. After an initial briefing, all participants provided their informed consent.

### Description of the inertial measurement cluster

**Fig. 3:**
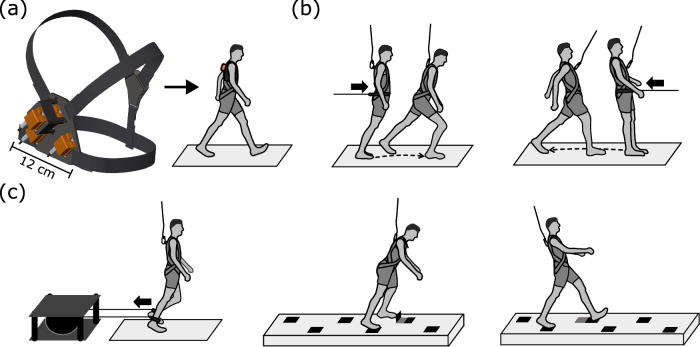
Schematic illustration of the inertial measurement cluster (IMC) and the applied perturbation paradigms. The model in (**a**) shows the IMC's attachment to the participant’s trunk using adjustable straps. The Xsens MTi-20 VRU IMUs were mounted on a rigid base and are represented by the orange boxes (**a**). Loss of balance in anterior or posterior directions during standing was initiated via a sudden release from a static, inclined position (lean-and-release task (**b**)). The inclination was maintained by a supporting cable horizontally attached to a belt around the participant’s pelvis, with the other end connected to a pneumatically driven brake-and-release system. During walking, we provided unpredictable trip- and slip-like perturbations (**c**). These perturbations were introduced by a pneumatically driven brake-and-release device with Teflon cables attached to the ankles, or by electronically triggered tripping and slipping elements (represented by the black elements (**c**)). The tripping element was activated upon touchdown of the trailing leg, while the anterior shift (slipping) was initiated upon touchdown of the perturbed leg onto the respective frame. For all tasks, the perturbation magnitudes varied from moderate to strong by adjusting the gait velocity (trips and slips) or changing the body’s inclination (lean-and-release task). Safety harnesses were worn during all tasks to prevent any part of the body, except the feet, from contacting the ground.

The characteristics of the IMC prototype have been described previously in Gießler et al.^18^. For this study, the cluster was further miniaturised and optimised for human locomotion analyses (Fig. 3a). To briefly summarise the IMC unit: it is based on four Xsens MTi-20 VRU (Movella Inc., Netherlands), each consisting of a 3D gyroscope and 3D accelerometer sensor packages. The sensor packages directly measure the linear acceleration vectors ***a***_*i*_ and the angular velocity vector ***ω***_*i*_ for *i* = 0, 1, 2, 3. Next, the relative vectors ***r***_*i*0_, pointing from the sensor packages *i* to 0 for *i* = 1, 2, 3, were approximately pairwise orthogonal.

Consequently, the underlying requirement outlined in Gießler et al.^18^ for linearly independent vectors hold. By interpreting the vectors as their coordinate matrices referenced in the IMC’s non-inertial and orthogonal measurement coordinate frame, the coordinates *α*_*k*_ for *k* = *x*, *y*, *z* of $\dot{{{{\boldsymbol{\omega }}}}}={\alpha }_{k}\,{{{{\boldsymbol{e}}}}}_{k}$ can be calculated with

1 $$\left(\begin{array}{c}{\alpha }_{x}\\ {\alpha }_{y}\\ {\alpha }_{z}\end{array}\right)={\left(\begin{array}{c}{\left({{{{\boldsymbol{r}}}}}_{10}\times {{{{\boldsymbol{r}}}}}_{20}\right)}^{T}\\ {\left({{{{\boldsymbol{r}}}}}_{20}\times {{{{\boldsymbol{r}}}}}_{30}\right)}^{T}\\ {\left({{{{\boldsymbol{r}}}}}_{30}\times {{{{\boldsymbol{r}}}}}_{10}\right)}^{T}\end{array}\right)}^{-1}\left(\begin{array}{c}{({{{{\boldsymbol{a}}}}}_{1}-{{{{\boldsymbol{a}}}}}_{0}-{{{\boldsymbol{\omega }}}}\times ({{{\boldsymbol{\omega }}}}\times {{{{\boldsymbol{r}}}}}_{10}))}^{T}\,{{{{\boldsymbol{r}}}}}_{20}\\ {({{{{\boldsymbol{a}}}}}_{2}-{{{{\boldsymbol{a}}}}}_{0}-{{{\boldsymbol{\omega }}}}\times ({{{\boldsymbol{\omega }}}}\times {{{{\boldsymbol{r}}}}}_{20}))}^{T}\,{{{{\boldsymbol{r}}}}}_{30}\\ {({{{{\boldsymbol{a}}}}}_{3}-{{{{\boldsymbol{a}}}}}_{0}-{{{\boldsymbol{\omega }}}}\times ({{{\boldsymbol{\omega }}}}\times {{{{\boldsymbol{r}}}}}_{30}))}^{T}\,{{{{\boldsymbol{r}}}}}_{10}\end{array}\right),$$

where ***ω*** was extracted as the mean value of ***ω***_*i*_ for *i* = 0, 1, 2, 3. Note that the inverse matrix in Eq. (1) always exists due to the linear independence of the vectors ***r***_*i*0_. Here, ***e***_*k*_ are the basis vectors of IMC’s non-inertial and orthogonal measurement coordinate frame. A more detailed description is shown in Supplementary Note 4 or in Gießler et al.^18^ Eqs. (1)–(7). To mitigate the impact of inherent stochastic noise in ***a***_*i*_ and ***ω*** in solving the coordinates *α*_*k*_, we applied the sensor fusion algorithm proposed in Gießler et al.^18^.

The relative vector norms of the miniaturised IMC were as follows: *r*_10_ = 0.08 m, *r*_20_ = 0.08 m and *r*_30_ = 0.05 m. The IMC had an approximate weight of 0.42 kg. Power supply as well as the data acquisition and computing units were decoupled from the sensor unit and fixated by means of adjustable straps at waist level. Raw angular velocity and linear acceleration vector measured by the Xsens MTi-20 VRU inertial sensors were processed on a microprocessor at each sample point to evaluate the mean angular velocity and the angular acceleration vector. To reduce deterministic errors in the IMC measurements, the misalignments between the sensor packages *i* to 0 were calibrated by static measurements in the gravitational field. The resulting vector coordinates were stored locally. To ensure system autonomy, no network connection was required. The custom-developed framework and its subroutines were optimised for low-power microprocessors. Upon providing consent, the custom-made IMC was centrally attached to the participant’s back (the upper edge of the IMC was levelled to the thoracic vertebras 3–4). Adjustable straps aided the attachment and proper fixation to specific anthropometrics of the participant’s trunk (Fig. 3a), minimising relative movement between the IMC and the trunk. This allowed the measured kinematics of the IMC to represent the trunk kinematics at close approximation. During all unperturbed and perturbed locomotion tasks, the three-dimensional kinematics of the trunk were measured with the IMC.

### Perturbed locomotion and activities of daily living analyses

Ten participants were invited to a laboratory and exposed to trip-like perturbations triggered by cable pulls on their ankles. Throughout the protocol, they were secured by a safety harness to avoid contact with the ground (except for the feet). In addition to this, we measured sit-to-stand, pick-and-drop, walking, running and staircase locomotion, in- and outside of the laboratory without the means of a safety harness. The remaining eight participants came to a second laboratory and were exposed to tripping and slipping (safety harness applied), triggered by electronically activated elements on a wooden pathway. Further to this, we measured ADLs (mentioned above) in the same order at same repetitions indoors in a public building. Within each participant group, the order of the perturbations was also standardised, but the perturbed trials for each paradigm were randomly assigned (including wash-out trials) for each participant. The measurement duration for each participant was restricted to 90 min in order to mitigate discomfort and overload for participants. If the measurement duration exceeded, the remaining tasks were not tested.

The number of perturbations varied between tasks due to various constraints and participants’ comfort. Fewer balance losses in the posterior direction during backward leaning tasks were performed since the posterior positioning of the ankle joint’s axis of rotation in relation to the antero-posterior foot distance limited the range of perturbation magnitudes for posteriorly directed falls. Furthermore, we implemented two different setups for trip-like perturbations to reflect better the greater variance in anterior perturbations during walking (perturbation of early or late swing phase leading to different balance recovery responses, i.e. lowering or elevating strategy), whilst slip-like perturbations were conducted using only one experimental setup. Lastly, some participants reported discomfort during posterior perturbations due to restricted visual feedback of foot placement, which sometimes led to early termination or rejection of the task (especially for higher posterior inclination angles), both resulting in more anteriorly directed than posteriorly directed perturbation trials that were recorded and analysed.

#### Trip- and slip-like perturbations during walking

Participants walked on a custom-built flat wooden negotiation (8 m length, 1.5 m width) which incorporated sudden electronically triggered trip and slip elements (Fig. 3c). All subjects returned to the initial position upon arrival at the end of the walkway, as both perturbations were triggered only in one direction. Trip-like perturbations were achieved through a frame integrated into the walkway, which would flip up, while slipping was facilitated by an incorporated frame shifting anteriorly. Both mechanisms were automatically triggered during random walking trials and at an initially unknown location of the walkway. Tripping elements were activated upon touchdown of the trailing leg on the proceeding element, whereas the anterior shift (slipping) was initiated upon touchdown of the perturbed leg onto the respective frame. To assess touchdown and automatically trigger slip- and trip-like perturbations, we used contact sensors integrated in each element. For each of the two perturbation paradigms (slipping and tripping), two trials at preferred and two trials at slower than preferred gait speed were performed and analysed, providing a variation in perturbation magnitude. To further vary trip-based balance disturbances we additionally applied posteriorly directed pulls on the swing leg during walking^27,28^. Subjects had to walk on a custom-built flat wooden walkway (8 m length, 1.5 m width), with Teflon cables attached by straps to both ankles. The cables were attached to a custom-built pneumatically driven brake-and-release device located behind the walkway (Fig. 3c). Analogue to the previous perturbation tasks, participants walked at two different gait speeds (preferred and slower than preferred). Once they arrived at the end of the walkway, they were guided back to the initial position to prevent tangling of the feet due to the Teflon cable. Thus, only one direction of movement was considered for measurements. Four to eight trip-like perturbations were induced at random forward-walking trials for each participant, with wash-out trials in between to prevent predictions. The perturbations were operated by means of a hand trigger connected to the perturbation device and evoked by a braking action of the Teflon cable on the left leg—during the mid-stance phase of the right foot and released at touchdown of the left foot. The perturbations were neither practised in advance nor announced immediately before exposure to provide novel and unpredictable perturbations.

#### Antero-posterior loss of balance during quiet stance

Similar to our previous studies^29^, we induced antero-posterior fall initiations during quiet stance using a lean-and-release task. Initially, participants were forward- or backward-inclined at various inclination angles, with their feet placed flat. Each participant performed six to eight anteriorly and three to four posteriorly directed sudden balance disturbances from static standing in random order. For both directions, the inclination was maintained by means of an inextensible, horizontally supporting cable attached to a belt around the participant’s pelvis. The other end of the cable was attached to a custom-built pneumatically driven brake-and-release system. Once the subject presented a stable position, the perturbation was initiated through a sudden release of the cable within 10–30 s (Fig. 3b). Subjects were instructed to recover balance using a single step. Nevertheless, the variation of inclination angles from moderate to high elicited different perturbation magnitudes and thereby provoked single-step strategies as well as multiple-step strategies^29^. Additionally, all perturbations are summarised and provided with concise descriptions in Supplementary Table S2.

#### Activities of daily living

To simulate a wide range of ADLs, our experimental setup comprised various locomotor tasks including unperturbed overground walking with three different speeds (preferred, slower and faster than preferred) as well as running at preferred speed, ascending as well as descending staircases, sit-to-stand and stand-to-sit and pick-up and drop of an object. For all walking and running conditions, measurements of trunk kinematics were taken over a walking distance of 12 m. For ascending as well as descending staircase locomotion, we used standardised staircase dimensions located in a public building (Length/Width/Height: [130/27/18] cm). Measurements were taken while subjects walked up or down over one floor (10 staircases). For sit-to-stand and stand-to-sit task measurements, all participants started and ended seated on a chair (Height: 0.46 m). Upon start, they stood up, walked at preferred speed over a 12 m pathway, turned around and returned to the chair. Furthermore, we analysed trunk kinematics during a pick-and-drop task. To incorporate a wider range of trunk flexion and extension movements in our ADLs, all participants were asked to grab a 5 kg-weighted box from the floor from an initially quiet and upright bipedal stance, followed by carrying the box at preferred gait speed over 5 m, safely drop the box on the floor and return. During all ADLs tasks (except sit-to-stand), the measurements of participants started and ended in quiet bipedal stance. All tasks were performed three times for each participant, with up to 3 min of rest between trials. Additionally, the acquired movements are summarised and provided with concise descriptions in Supplementary Table S2.

#### Baseline curves

Baseline curves were established based on three separate walking trials performed by each participant on a wooden walkway after a familiarisation period. The participants were not connected to the safety harness and walked unperturbed for a distance of 12 m at a target speed of  ≈1.4 m/s. The measurement began 3 s prior to the initiation of walking and ended 3 s after the participants stopped walking. These baseline trials were excluded from subsequent analyses and were not considered in the results’ evaluation.

### Framework to detect and classify balance disturbances among locomotion tasks

**Fig. 4:**
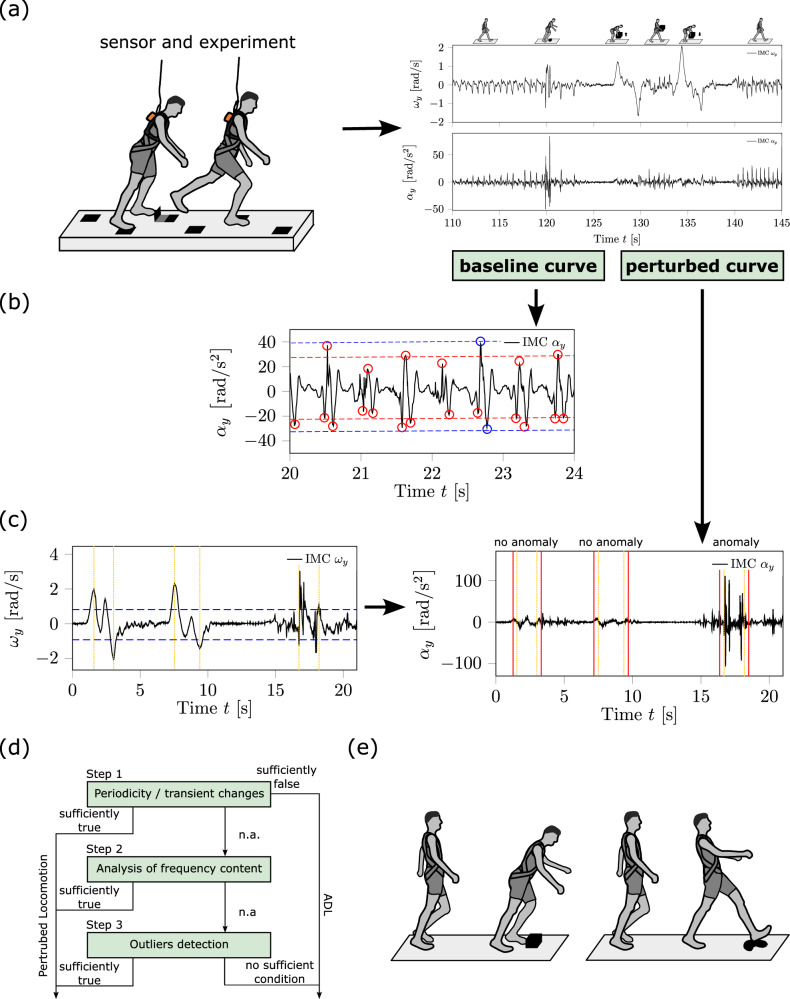
Schematic overview of the automated framework for detecting and distinguishing balance recovery responses. This schematic outlines the step-by-step process of the proposed framework designed to detect anomalies in trunk kinematics, with a focus on identifying balance recovery responses to perturbations and classifying the type of perturbation. Step (**a**) shows the remote monitoring of the trunk kinematics using the wearable inertial measurement cluster (IMC) placed on the participant’s trunk during locomotion tasks that include both normal activities of daily living (ADLs) and externally applied perturbations (e.g. trips, slips). Step (**b**) is the automated determination of personalised thresholds to take into account individual gait characteristics. In Step (**c**), the observation windows were detected based on the personalised thresholds and a simultaneous analysis of the trunk angular velocity and acceleration vector coordinate along the transverse axis, identifying time periods containing potential anomalies (necessary condition). In Step (**d**), the observation windows were analysed for frequency content, periodicity and outliers to assess whether the detected anomaly indicates a sufficient condition for a balance perturbation. Finally, in Step (**e**), the framework classifies the locomotor task and the type of perturbation, identifying the specific perturbation types.

In our previous study^18^, we developed an automated framework designed to detect anomalies in trunk kinematics resulting from externally induced trip-like perturbations, with a particular emphasis on identifying fall initiation, balance recovery performance and adaptation phenomena. Herein, this framework was enhanced to automatically detect balance recovery responses to various perturbations and to distinguish these from ADLs. Additionally, it can classify the type of perturbation (i.e. tripping vs. slipping vs. anterior loss of balance during standing vs. posterior loss of balance during standing). The framework’s primary structure includes a simultaneous analysis of trunk angular velocity and acceleration vector coordinate along the transverse axis in the trunk’s principal axis system (*y*-axis; directly measured via IMC). Personalised thresholds were automatically extracted to resolve and consider individual gait characteristics. The concept of necessary and sufficient conditions for detecting balance recovery responses was introduced to distinguish between perturbed locomotor and ADLs. Indicators for these conditions were extracted through the subsequent data analysis. The steps of the framework are outlined schematically in Fig. 4, providing a visual summary of the structure of the framework from trunk kinematics measurement to loss of balance and classification of perturbation types. During the framework’s setup, the dataset was divided into a setup dataset (four participants) and a validation dataset (14 participants) that remained untouched during development. The entire dataset of 18 participants was used for the final evaluation of the framework’s metrics. Measurement trials where interference from the safety harness cable introduced artefacts into the kinematic signal were discarded.

#### Definitions to recognise balance recovery responses following perturbations

**Fig. 5:**
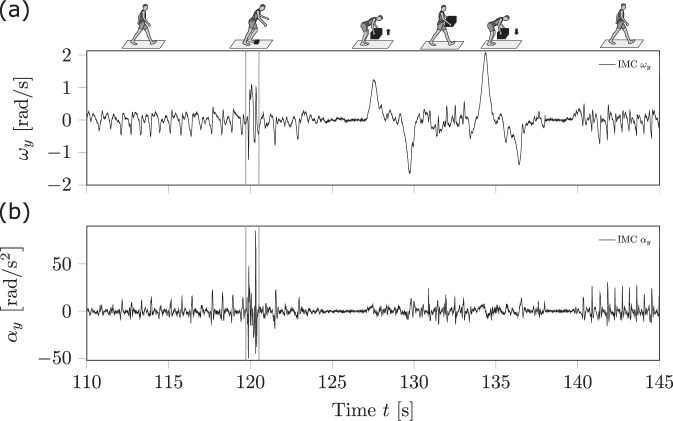
Framework’s results of the automatically detected balance recovery response in a typical locomotion sequence of daily life. In (**a**), the raw data of the trunk’s angular velocity *ω*_*y*_ and in (**b**), the angular acceleration component *α*_*y*_ around the transversal axis (*y*-axis) are shown for perturbed walking, pick-and-drop task and unperturbed walking. The corresponding locomotion task is indicated with the stick figures on top of the raw data section. The black vertical lines highlight the automatically detected balance recovery response. Anomalies appeared in *ω*_*y*_ for both perturbed locomotion and typical activities like object manipulation. However, by jointly analysing the trunk’s *ω*_*y*_ and *α*_*y*_ components, it became clear that only perturbed locomotion exhibits anomalies in both kinematic signals. Note that during time frame ≈ 110–115 s, the gait velocity was lower in relation to the time frame ≈ 140–145 s leading to different signal characteristics.

In this study, a near-fall was defined as an event in which a sudden loss of balance occurs (i.e. caused by a perturbation) and was successfully recovered by a balance recovery response. Therefore, we based this definition on anomalies in the magnitude and frequency spectrum of the trunk’s kinematic curve path. To generally distinguish a near-fall event from falls and negligible trunk kinematics related to potential balance recovery responses, we introduced lower and upper boundaries (cf. Fig. 5). Since this work focused on detecting balance recovery responses, regardless of whether balance recovery or a fall occurred, defining the lower boundary was essential.

**Fig. 6:**
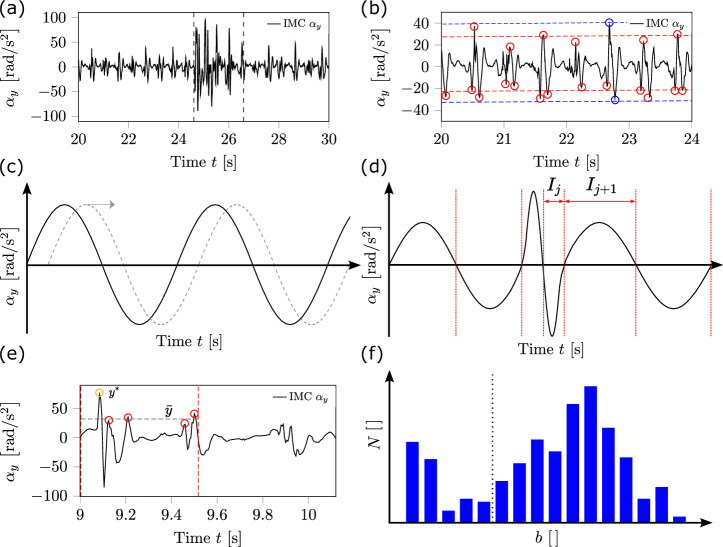
Schematic illustration of trunk kinematics and the subroutines. In (**a**) and (**b**), the perturbed and unperturbed trunk angular acceleration about the transverse axis *α*_*y*_ are represented, respectively. In (**c**–**f**), the subroutines for the data processing used to automatically detect balance recovery responses are visualised. In (**a**), one result of an automatically detected balance recovery response for a trip is illustrated. In (**b**), the global extrema and specific local maxima of an unperturbed baseline walking are shown (blue and red circles, respectively; where the local maxima are a consequence of a touchdown). In **c**, the method to calculate the autocorrelation of the same measurement trial and in **d**, an exemplary segmentation of the kinematics curve path for the local extrema identifier algorithm is represented. In (**e**), method 2 for the outlier identification is shown for a given subset *T*, and **f** represents a histogram of the peak distributions analysis.

In general, to classify a temporal bounded observation window in the trunk kinematics as a balance recovery response potentially corresponding to a near-fall event, we applied the concept of necessary and sufficient conditions. A necessary condition herein was the detection of an observation window. An observation window itself was defined so that its time interval must contain both kinematic signal anomalies regarding the curve’s amplitudes. Anomalies in trunk kinematics were defined as the exceedance of a predefined ratio of the local maxima and minima for the trunk angular velocity and angular acceleration signal compared to the respective global maxima found in unperturbed baseline curves (Fig. 6b). Whereas sufficient conditions were related to kinematics amplitudes with outlier characteristics or transient irregular frequency content within the trunk kinematics curve paths enclosed by the observation window (Fig. 6a). Finally, if one of the sufficient conditions was recognisable, this observation window was assigned as a balance recovery response.

To detect anomalies in trunk kinematics, we applied personalised thresholds to consider individuals’ gait characteristics derived from the individual’s baseline curves (unperturbed walking with a target velocity of  ≈1.4 m/s). In a first step, we defined local extrema as the interval-wise maximum or minimum bounded through two zero crossings of a signal. Accordingly, the global extreme was the largest local maximum or smallest local minimum in the entire measurement series (blue dots in Fig. 6b). For unperturbed baseline, there were specific local maxima in the angular acceleration signal identified as a direct consequence of a touchdown of the respective foot (marked in red in Fig. 6b). In a pilot study, the validity of the proposed relation (specific local maxima induced by foot’s touchdown) was confirmed by comparing our obtained frequency of the specific local maxima by the autocorrelation analysis (cf. section ‘Autocorrelation’) for gait speed of  ≈1.4 m/s with the walking cadence of 21–40 years old adults for a gait speed of 1.34 m/s analysed in Tudor-Locke et al.^30^. The averaged steps per minute values for all participants obtained by our autocorrelation analysis was $116.8\pm 7.7\,{{{\rm{steps}}}}/\min$ compared to $113.6\pm 6.1\,{{{\rm{steps}}}}/\min$ in Tudor-Locke et al.^30^. Consequently, we extracted the personalised thresholds so-called averaged local maxima (indicating touchdown consequences in the trunk kinematics) and minima, as well as the global maximum and minimum. To identify an individual’s time-related gait characteristics from the baseline curve, we defined a step as a sequence of events and phases beginning with the heel strike event of one foot and ending with the heel strike event of the contralateral leg. The periodic frequency of the touchdown-induced local maxima, thus the frequency of the touchdown events of one of the two feet, was defined as the frequency (so-called averaged step frequency).

An example of the proposed procedure is provided in Fig. 5, demonstrating the relevance of assessing both the trunk angular velocity and acceleration coordinates of the transverse axis in a joint analysis to accurately detect and distinguish antero-posterior near-falls from ADLs. When considering only the transverse axis component of the trunk’s angular velocity, anomalies occur in perturbed locomotion, as well as in typical ADLs such as picking up and dropping objects (Fig. 5a). Instead, perturbed locomotion is characterised by anomalies in both trunk kinematics signals (Fig. 5a, b).

### Subroutines for data processing

In the following, we present the underlying subroutines utilised in the kinematics data analyses, i.e. threshold determination, observation window detection and subsequent data analyses. The subroutines are stand-alone algorithms with predefined inputs and outputs, and were used at different points and/or combinations throughout the framework.

In general, for all subroutines the following applies. The discrete sample points *t*_*i*_ form a non-empty subset *I* (domain; timeline of the measurement series) with $I\subset {\mathbb{R}}$. Let *f*: *I* → *M*, *t* ↦ *f*(*t*) be a mapping giving the measured kinematic value *f*(*t*_*i*_).

#### Autocorrelation

To identify periodically occurring patterns and their corresponding frequencies in a measurement series, we used autocorrelation analysis for time-discrete function *f*, e.g. to identify the participant’s averaged step frequency for preferred walking. The autocorrelation coefficient, *c*_*k*_, between *f* and a time-shifted variant of *f* (Fig. 6c) was quantified using

2 $${c}_{k}= {\sum}_{i=1}^{T-k}(f({t}_{i+k})f({t}_{i})).$$

Here, *f*(*t*_*i*_) is the measurement value at the corresponding sample time *t*_*i*_, *k* is the constant temporal offset to the original measurement series, and *T* describes the number of sample points. Further, *c*_*k*_ was calculated for predefined values of *k* for *k* = *n*Δ*t* and *n* = 0, …, *N*, where Δ*t* is the time equivalent of the sample rate of the measured series. We normalised the result by

3 $${r}_{k}=\frac{{c}_{k}}{{c}_{0}},$$

where *c*_0_ corresponds to the result of *k* = 0. The temporal offsets *k* of local maxima in *r*_*k*_ were identified and further used in the framework for subsequent analyses.

#### Local extrema identifier

Next, we employed the so-called local extrema identifier algorithm to analyse specific local extrema of *f*. First, *I* is partitioned into non-empty subsets *I*_*j*_ for *j* = 1, …, *J*. The starting points of *I*_*j*_ for *j* = 2, …, *J* are defined by a sign change of the kinematic value *f*(*t*_*i*_) and *f*(*t*_*i*+1_) at the adjacent elements *t*_*i*_ and *t*_*i*+1_, using

4 $$\varpi (f({t}_{i})) \, \ne \, \varpi (f({t}_{i+1}))$$

where *t*_*i*_ < *t*_*i*+1_ holds and *ϖ* is defined as

5 $$\varpi :{\mathbb{R}}\to \{-1,1\}$$

$$x \, \mapsto \left\{\begin{array}{cc}-1: & x \, < \, 0 \\ \, \, \, \, 1: & x \ge 0 \end{array}\right.$$

The respective endpoint of *I*_*j*_ for *j* = 1, …, *J*−1 is the element immediately prior the next start point of *I*_*j*+1_ (Fig. 6d). The starting point for *I*_1_ is *t* = 0 and the endpoint for *I*_*J*_ is the last sample point, i.e. $\max (I)$. Hence, all elements in the *j*th image *M*_*j*_ = *f*(*I*_*j*_) are either exclusively non-negative or exclusively negative. Next, let $\hat{K}$ and $\check{K}$ be subsets defined by

6 $$	 \hat{K} =\left.\right\{(y,x)\in M\times I\,| \,\exists j\in \{1,2,...,J\}: \\ 	 \left.\right(y =\max ({{{\rm{abs}}}}({M}_{j}))\wedge \hfill \\ 	 x =\min \left(\left\{x\in {f}^{-1}({M}_{j})\,| \,y=f(x)\right)\right\}$$

7 $$	 \check{K} =\left.\right\{(y,x)\in M\times I\,| \,\exists j\in \{1,2,...,J\}:\\ 	 \left.\right(y =-\max ({{{\rm{abs}}}}({M}_{j}))\wedge \\ 	 x =\min \left(\left\{x\in {f}^{-1}({M}_{j})\,| \,y=f(x)\right)\right\}$$

with the range of the absolute value function

8 $$\begin{array}{r}{{{\rm{abs}}}}(M)=\{x\in {\mathbb{R}}| \,\exists e\in M:x=| e| \}\end{array}$$

for any domain $M\subset {\mathbb{R}}$.

Subsequently, we evaluated the separate mean values $\bar{\hat{m}}$ and $\bar{\check{m}}$ with

9 $$\bar{\hat{m}}=\frac{1}{| \hat{K}| } {\sum}_{(y,x)\in \hat{K}}y$$

10 $$\bar{\check{m}}=\frac{1}{| \check{K}| } {\sum}_{(y,x)\in \check{K}}y,$$

where ∣*M*∣ denotes the number of elements in a finite set *M*, and the global maximum ${\hat{m}}_{{{{\rm{global}}}}}=\max (\{y\in M| \,\exists x\in I:(y,x)\in \hat{K}\})$ and minimum ${\check{m}}_{{{{\rm{global}}}}}=\min (\{y\in M| \,\exists x\in I:(y,x)\in \check{K}\})$. For example, the mean values and the global maximum and minimum were used as the personalised averaged local or global thresholds, respectively.

#### Outlier identification

Further, we considered two different principles for the identification of outlier measurements in a given subset $T=A\times B\subset {{\mathbb{R}}}^{2}$ with (*y*, *x*) ∈ *T*. Here, *x* is the sample point and *y* is the measured kinematic value, which can be assumed either non-negative or strictly negative. Since the values *y* ∈ *A* cannot be assumed to be normally distributed we used quartiles for the outlier identification. More specifically, for outlier identification method 1, we classified *y* as an outlier if it was more than the interquartile range scaled by the predefined factor *c* above the upper quartile (75%) or below the lower quartile (25%) (default: *c* = 1.5).

In outlier identification method 2, first, we identified (*y*, *x*)^*^ ∈ *T* whose first entry *y* is the absolute maximum y^*^, defined by ${y}^{* }=\max (\{a\in {{{\rm{abs}}}}(A)| \,\exists (y,x)\in T:a=| y| \})$. Second, we formed T^*^ with T^*^ = *T*⧹{(*y*, *x*)^*^}. Subsequently, the mean

11 $$\bar{y}=\frac{1}{| {T}^{* }| } {\sum}_{(y,x)\in {T}^{* }}| y|$$

was calculated. Lastly, y^*^ was identified as an outlier if

12 $${y}^{* }\ge c\,\bar{y},$$

held (Fig. 6e). Here, *c* was also predefined factor, with a default value of *c* = 1.5.

#### Local extrema distribution monitor

Finally, we used the so-called local extrema distribution monitor by utilising two histograms, one for the identified local maxima represented by all *y* of $(y,x)\in \hat{K}$ and one for local minima represented by all *y* of $(y,x)\in \check{K}$, to check their distribution and, based on this, to assess whether there were irrelevant magnitudes, respectively (Fig. 5f). For the histogram, bins *b* for *b* = 1, …, *B* with an equidistant and predefined bin width *h* and the bin interval *i*_*b*_ = [*f*_*b*,__low_, *f*_*b*,__up_] were considered, where *f*_*b*,__low_ was the lower, *f*_*b*,__up_ the upper bound and, *N*_*b*_ was the bin size of bin *b*.

We compared *N*_*b*_ of the bins for *b* = 1, …, 5 to identify if there were one or more bins with outlier characteristics (according to the quartiles definition also used in Outlier identification method 1; Fig. 6f). Provided that at least one *N*_*b*_ could be assigned as an outlier, the highest upper bound *f*_*b*,__up_ for maxima and the lowest lower bound *f*_*b*,__low_ for minima define the irrelevant extreme magnitude *f*_irr,_ _max._ as well as *f*_irr,_ _min._, respectively, which were declared as irrelevant. In addition, these magnitudes were subsequently limited by ${f}_{{{{\rm{irr}}}},{{{\rm{max.}}}}}=\max ({f}_{{{{\rm{irr}}}},{{{\rm{max.}}}}},\,2\,{{{\rm{rad}}}}/{{{{\rm{s}}}}}^{2})$ considering the identified local maxima and ${f}_{{{{\rm{irr}}}},{{{\rm{min.}}}}}=\min ({f}_{{{{\rm{irr}}}},{{{\rm{min.}}}}},\,-2\,{{{\rm{rad}}}}/{{{{\rm{s}}}}}^{2})$ considering identified local minima. The respective bound (±2 rad/s^2^) was applied since this range of trunk angular acceleration can be mainly attributed to signal noise-induced local extrema or irrelevant trunk motion in terms of balance recovery response detection.

### Personalised threshold determination

Three unperturbed baseline curves were used to calculate the personalised thresholds. In this study, the ground surface varied between a wooden surface (used for perturbed trials and some unperturbed walking trials as ADLs) and different types of ground surfaces (used for ADLs). We used data from baseline walking measured on the wooden surface. All personalised thresholds were automatically extracted and calculated globally (once before the perturbed trials started).

The required personalised thresholds included the averaged step frequency, and the averaged local and the global maxima and minima. The autocorrelation analysis was used to determine the averaged step frequency, while the global extrema were identified using the extrema identifier routine. To calculate the averaged local maxima and minima, we employed a combination of the extrema identifier and the distribution monitor (cf. section ‘Subroutines for data processing’; slightly adjusted to Gießler et al.^18^). For the averaged local extrema calculation, the objective was to exclude irrelevant magnitudes, such as those caused by signal noise in low magnitude ranges, which do not represent trunk kinematics. Therefore, in a preliminary step, the distribution monitor and outlier detection identified and excluded potential irrelevant magnitudes within the bins ${b}_{{\alpha }_{y}}$ and ${b}_{{\omega }_{y}}$, using limits of  ±5 rad/s^2^ and  ±0.25 rad/s. Bin widths were set at ${h}_{{\alpha }_{y}}=1\,{{{\rm{rad}}}}/{{{{\rm{s}}}}}^{2}$ and ${h}_{{\omega }_{y}}=0.05\,{{{\rm{rad}}}}/{{{\rm{s}}}}$ based on pilot study results. An iterative routine was then applied to identify relevant local maxima, primarily induced by foot touchdowns, until the time difference between identified adjacent maxima matched the averaged step frequency (±10%). A similar method, but only with two iterations, was used for local minima, as their occurrence was unrelated to periodic or specific gait events.

### Observation window determination

The algorithm for determining the onset and offset of the observation window was based on our previous framework^18^, but enhanced for improved precision, particularly in onset and offset detection. The accurate onset detection was critical for analysing preceding locomotion and classifying perturbation types. The following adjustments were implemented compared to the previous framework^18^.

**Fig. 7:**
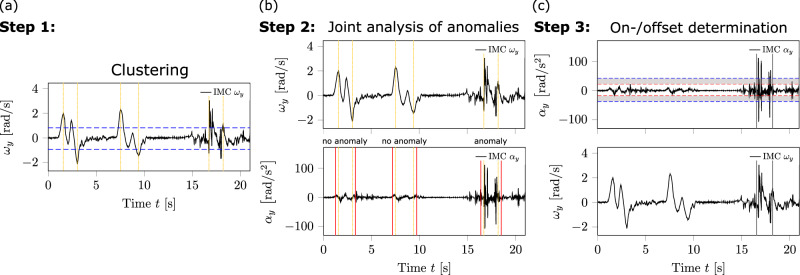
Schematic illustration of the procedure to automatically determine observation windows. The procedure is divided into three steps (**a**–**c**), based on joint analysis to identify anomalies in the trunk kinematics. In (**a**), the detected anomalies in the trunk’s angular velocity component around the transverse axis *ω*_*y*_ are clustered in temporary observation windows. An anomaly was defined as personalised threshold exceedance. In **b**, the combined analysis of *ω*_*y*_ and the trunk’s angular acceleration component around the transverse axis *α*_*y*_ is shown. In this step, we check whether there are anomalies in *α*_*y*_ present in the extended observation windows (red vertical lines). In (**c**), the perturbation onset and the offset of the balance recovery response are determined for all extended observation windows containing anomalies in both kinematics, indicated by grey vertical lines. The offset determination takes into account the displayed threshold bandwidths.

Observation windows containing perturbed locomotion were identified if at least one local maximum or minimum in both angular velocity and angular acceleration curves exceeded 1.15 times the personalised threshold of global maximum and/or minimum, respectively (represented with blue horizontal lines in Fig. 7a). The sensitivity for the observation window detection could be adjusted by modifying this ratio. Local extrema exceeding these thresholds in the angular velocity signal were clustered into temporary observation windows based on time differences between adjacent extrema (yellow lines in Fig. 7a). Clusters were separated if the adjacent extrema were temporally separated by more than the time equivalent of the step frequency.

Next, each temporary observation window was checked for extrema in the angular acceleration signal exceeding the same ratio. For this check, an observation window was extended by half the time equivalent of the averaged step frequency on both sides (red vertical lines in Fig. 7), called temporary observation window. Observation windows containing anomalies in both kinematics were then forwarded for onset and offset analyses (Fig. 7b).

The onset of an observation window was defined as the time point immediately after the first zero crossing in the angular acceleration signal that precedes the first extreme within the temporary observation window. The offset was determined by analysing the convergence of local extrema magnitudes relative to the threshold bandwidths (Fig. 7c). Specifically, the offset was set at the subsequent zero crossing of the last local extreme with respect to the time within the temporary observation window. Additionally, subsequent local extrema had to remain within or below the threshold bandwidths for a time equivalent to the step frequency (grey shaded area; onset and offset marked by grey lines in Fig. 7c). If any extreme exceeded the threshold during this interval, the temporary observation window was extended to include this extreme, and the offset detection routine was repeated.

### Subsequent data analysis to distinguish between perturbed motion and ADLs

**Fig. 8:**
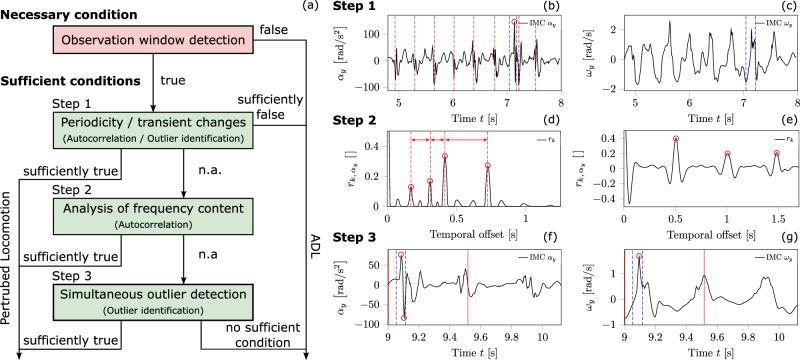
Schematic illustration of the subsequent data analysis. The flowchart of the subsequent data analysis is represented in (**a**) and the underlying steps to classify an observation window as a balance recovery response or daily activity in (**b**–**g**). The subsequent data analysis was divided in three steps. In each step, we analysed the detected observation window (necessary condition) and determined whether it contained additional predefined criteria (i.e. sufficient condition). The curve sections displayed in (**b**, **c**) represent a sample observation window identified for a running trail. Further, **b**, **c** show the analyses regarding periodic pattern or transient irregular frequency content including anomalies with outlier characteristics (step 1) for the coordinates around the transverse axis of the trunk’s angular acceleration *α*_*y*_ and velocity *ω*_*y*_. Considering step 2, **d** shows a schematic of irregular frequency content evaluated by the autocorrelation coefficient peaks ${r}_{k,{\alpha }_{y}}$ of a balance recovery response, where **e** shows an expected (regular) frequency content with recognisable periodic patterns of unperturbed walking. In **f**, **g**, the trunk kinematics *α*_*y*_ and *ω*_*y*_ for the same balance recovery response are shown. The red lines indicate the identified observation window, the blue dotted lines indicate the time window (±0.15 s) centred around the local maxima peak with outlier characteristic. In **g**, the same time window is highlighted (blue dotted lines), i.e. the analysis of whether there also exists an outlier (step 3).

To classify a motion as perturbed or a consciously performed ADL task, identifying an observation window was necessary but not sufficient. For example, running or picking up an object often generates in higher magnitudes of the extrema in the trunk angular kinematics than normal walking. Thus, additional criteria were required to confirm a balance recovery response within the observation window based on the subroutines (cf. section ‘Subroutines for data processing’; Fig. 8a).

In step 1, we simultaneously checked the observation window for both periodicity as well as transient frequency changes greater than the averaged step frequency, caused by at least one outlier according to outlier identification method 2 (Fig. 8b, c). Within the observation window, periodic occurrence repetitions were identified by examining the autocorrelation and the time differences between adjacent local maxima in the angular acceleration signal, i.e. the touchdown-induced local maxima. If three or more periodic repetitions were identified along with a similar occurrence frequency, this indicated a sufficient condition and the motion within the observation window was classified as unperturbed ADL. If irregular transient frequency content caused by time-correlated local extrema outliers (outlier identification method 2) in both the angular velocity and angular acceleration signals was identified, it indicated a sufficient condition for a balance recovery response. Time-correlated outlier characteristics mean that an identified angular velocity outlier had to show an evident temporal correlation to an angular acceleration outlier.

For example, the curve sections displayed in Fig. 8b, c represent a sample observation window detected for a running trail. The red dotted vertical lines in Fig. 8b indicate the identified periodic pattern of the angular acceleration signal. In the time interval, marked by the blue dotted vertical lines in Fig. 8b, the angular acceleration signal contains a transient change in frequency caused by an outlier. The same time interval was used to check for outliers identifiable in the angular velocity signal (Fig. 8c). In Fig. 8c, there is no outlier identifiable in the respective time interval (blue vertical dotted lines); therefore, this movement was classified as unperturbed ADLs.

If step 1 did not provide a sufficient condition, step 2 analysed frequency content in both angular velocity and angular acceleration signals within the observation window extended by a 1.25 s offset using autocorrelation. The identified autocorrelation coefficient peaks of *r*_*k*_ were checked by outlier detection based on outlier identification method 1 to identify frequency content which lay above the averaged step frequency and may have a transient character (Fig. 8d). Detected outliers were examined with respect to their associated time shift *k* to identify transient characteristics or any repeated patterns, such as multiples of each other, as shown in Fig. 8e). For example, Fig. 8d shows transient frequencies higher than the averaged step frequency which do not repeatedly occur (sufficient condition), as compared to Fig. 8e. In comparison, Fig. 8e shows the frequency content of unperturbed walking.

If step 2 failed, step 3 examined outliers in both kinematic quantities within an extended observation window, including temporal boundary offsets—four times the averaged step frequency—before the onset and after the offset. This approach allowed for assessing differences in local maxima or minima magnitudes within the time intervals immediately preceding and following the observation window. Outlier detection followed method 2. Around the time points of the identified outliers, a centred time interval of  ±0.15 s with respect to the identified outlier time points was used to analyse the temporal correlation between angular velocity and acceleration outliers. For instance, in Fig. 8f, g, the identified outliers are marked with red circles. The observation window (without the additional temporal boundary offsets) is marked with red and time interval with blue dashed vertical lines. If correlation outliers were found in both curves, the motion was classified as perturbed; otherwise, it was categorised as unperturbed ADL.

### Differentiation of locomotor task before perturbations

To identify the motion type immediately preceding a perturbation, we analysed a specific time interval of the angular acceleration signal. The duration of the time interval was equivalent to four times the personalised threshold of the averaged step frequency and ended with the sample point immediately before the perturbation onset. We compared the deviation of the local extrema magnitudes and the periodicity of the angular acceleration signal to the baseline curves for standing, walking and running. We specifically examined the difference between the average local maximum value and the frequency of periodic patterns in the angular acceleration curve within the specified time interval, compared to baseline curves for standing, walking and running. To identify the average local maximum value and the frequency of periodic patterns, we applied the same principle as in the analysis concerning thresholds of a baseline curve and the autocorrelation (cf. sections ‘Local extrema identifier’ and ‘Autocorrelation’). Ultimately, the reference curve with the least deviation represented the behaviour that approximated the motion pattern prior to the perturbation.

### Distinguishment between types of perturbations

In general, human walking is inherently unstable, characterised by phases with at least one non-actuated degree of freedom (represented by the red arrow in Supplementary Fig. S3a–c). This implies that the kinematics of this degree of freedom cannot be controlled directly. Consequently, during the swing phase of one leg in forward locomotion, the human body rotates around a tipping edge of the base of support, in a manner that is not directly controllable. Therefore, mechanisms must be applied to indirectly control the kinematics of the non-actuated degree of freedom. In the absence of external perturbations, the under-actuated phase of the unperturbed step ends with the heel strike of the swinging leg, as the increase in the base of support allows the total ground reaction force to compensate for the rotation around the tipping edge (Supplementary Fig. S3a). However, in the presence of a trip- or slip-like perturbation during forward walking, the mechanism to decelerate the angular velocity of the non-actuated degree of freedom may be delayed. Consequently, a balance recovery response possibly involving the trunk must be applied. In this case, the trunk is rotationally accelerated in the antero-posterior direction, depending on the type of perturbation (trip or slip; Supplementary Fig. S3b or c). The angular acceleration of the trunk induces a moment of inertia related to the tipping edge, resulting in an opposite overall body angular momentum change. This reduces the angular velocity of the non-actuated degree of freedom. In quiet bipedal stance, a similar principle exists for balance recovery responses to compensate for a non-actuated degree of freedom caused by an impulsive force acting on the individual for a short but finite period. In a arbitrary inertial Cartesian coordinate system, where the *x*-axis points in the anterior direction and the *z*-axis points perpendicular to the ground, the balance recovery response of the trunk induces inertia moments around the tipping edge with different signs around the transverse axis. Thus, these balance recovery responses can also be resolved as local extrema in the trunk angular momentum and therefore in the trunk angular velocity component around the transverse axis.

To classify the type of perturbation accurately, the algorithm used the results of the analysis of the preceding movement type and examined the trunk angular velocity coordinate in the antero-posterior direction. Angular velocity was chosen as the evaluation parameter because of its robustness in interpreting local extrema. In trunk’s angular acceleration, local extrema can be induced by various mechanisms, such as a foot’s touchdown. Generally, a foot touchdown induces a local maximum over a short period of time with respect to the time points of the enclosing zero crossings, resulting in an irrelevant magnitude of a local extreme in the angular velocity signal compared to those induced by balance recovery responses. Therefore, it was more robust to attribute the local extrema to the initial balance recovery response behaviour compared to the them in the trunk angular acceleration.

To predict the perturbation type, the algorithm analysed the initial balance recovery response with respect to the angular velocity signal in a predefined time interval (half of the observation window duration, with an upper limit of 0.25 s). The type of perturbation was classified by considering the sign of identified angular velocity extrema and the preceding motion type. The differentiated perturbation types were trips, slips and loss of balance in anterior or posterior directions during quiet bipedal stance. More detailed information on the mechanical interpretation of the balance recovery response algorithm can be accessed in Supplementary Note 5.

### Statistics

Concerning the objective comparison of the IMC- and the IMU-framework combinations, we used statistical quality criteria of classification. Herein, we considered, firstly, the prevalence-independent statistics sensitivity and specificity. The sensitivity is defined as

13 $${p}_{{{{\rm{sens}}}}}=\frac{{r}_{{{{\rm{p}}}}}}{P},$$

where *r*_p_ are the true-positive detections and *P* is the total amount of perturbed trials defined as positives. Next, the specificity is defined as

14 $${p}_{{{{\rm{spec}}}}}=\frac{{r}_{{{{\rm{n}}}}}}{N},$$

where *r*_n_ are the true negative detections and *N* is the total amount of unperturbed trials defined as negatives.

In addition, we analysed the positive predictive value *p*_PPV_, which corresponds to the conditional probability that the trial was perturbed under the condition of a positive detection of the framework. It is defined as

15 $${p}_{{{{\rm{PPV}}}}}=\frac{{r}_{{{{\rm{p}}}}}}{{r}_{{{{\rm{p}}}}}+{f}_{{{{\rm{p}}}}}},$$

where *r*_p_ + *f*_p_ represents the total number of positive predictions.

Due to the knowledge whether a trial was perturbed or unperturbed, we calculated the study’s prevalence rate concerning perturbed locomotion as the ratio of perturbed to the unperturbed trials. Based on this, the *p*_PPV_ can also be calculated with

16 $${p}_{{{{\rm{PPV}}}}}=\frac{{p}_{{{{\rm{sens}}}}}\cdot {p}_{{{{\rm{prev}}}}}}{{p}_{{{{\rm{sens}}}}}\cdot {p}_{{{{\rm{prev}}}}}+(1-{p}_{{{{\rm{spec}}}}})\cdot (1-{p}_{{{{\rm{prev}}}}})},$$

cf. Bayes’ theorem, where *p*_prev_ corresponds to the prevalence rate. With Eq. (16), we extrapolated the *p*_PPV_ considering different prevalence rates. Finally, we considered the *F*_1_ score, defined as the harmonic mean of the positive predictive value and the sensitivity with

17 $${F}_{1}=\frac{2\,{p}_{{{{\rm{PPV}}}}}\cdot {p}_{{{{\rm{sens}}}}}}{{p}_{{{{\rm{PPV}}}}}+{p}_{{{{\rm{sens}}}}}}.$$

The resulting metrics for each evaluated approach are presented as individual data.

## Supplementary information

Supplementary File

nr-reporting-summary

## Data Availability

Datasets^31^ generated during and/or analysed during the current study are available on Figshare.

## Code availability

The code used for the framework (mathematical algorithms) to analyse the kinematic data generated during the current study is being made available by the corresponding author for research (non-commercial use) or on reasonable request.
