# Supplementary material for: A framework to automatically detect near-falls using a wearable inertial measurement cluster
Source: Commun Eng. 2024 Dec 16;3:181. doi: 10.1038/s44172-024-00325-x (PMC11649693; doi:10.1038/s44172-024-00325-x)
Supplement: Supplementary file 1 — Supplementary File [file 44172_2024_325_MOESM1_ESM.pdf]

A framework to automatically detect near-falls using a wearable inertial measurement cluster

Maximilian Gießler[1,2*], Julian Werth[2], Bernd Waltersberger[1] and Kiros Karamanidis[2,3]

[1*]Department of Mechanical and Process Engineering, Offenburg University of Applied Sciences, Offenburg, Germany.
[2*]Sport and Exercise Science Research Centre, School of Applied Sciences, London South Bank University, London, United Kingdom.
[3*]Department of Sport Science, Faculty of Mathematics and Natural Sciences, University of Koblenz, Koblenz, Germany.

*Corresponding author(s). E-mail(s): maximilian.giessler@hs-offenburg.de;

Supplementary information

Supplementary Note 1

Table S1 Comparison of Metrics between IMC and IMU base don the central difference quotient

Metric	IMC	IMU (CD)
Sensitivity	100 %	98.9 %
Specificity	98.4 %	92.4 %
Positive Predictive Value	96.2 %	83.9 %
F1-Score	98.1 %	90.8 %
Perturbation type detection	100 %	58.1 %
Locomotion type detection preceding perturbation	100 %	59.2 %

Supplementary Note 2

Table S2 Summary of activities in daily living (ADLs) and perturbed locomotion tasks executed form each participant.

Name	Description
Unperturbed overground walking	Walking – 12 m distance – three different speeds (preferred, slower, and faster than preferred)
Running	12 m distance – at a preferred speed
Ascending Staircase	Walking up – standardized staircase (10 steps, [130/27/18] cm dimensions)
Descending Staircase	Walking down – standardized staircase (10 steps, [130/27/18] cm dimensions)
Sit to Stand; Stand to Sit	Stand up – preferred speed walking over 12 m – turn around – returned to the chair
Pick and Drop	5 kg box – pick up from the floor – 5 m carrying – drop the box – return to start
Trip-like perturbation	Walking – 8 m walkway – sudden electronically triggered trip elements – triggered by leg's touchdown
Slip-like perturbations	Walking – 8 m walkway – sudden electronically triggered slip elements – triggered by leg's touchdown
Trip-like perturbation (cable pull)	Teflon cables attached to ankles – walking – pulled during mid-stance phase
Antero-posterior balance perturbation	Lean-and-release task – forward- or backward-inclined – various angles – sudden release to initiate fall

Supplementary Note 3

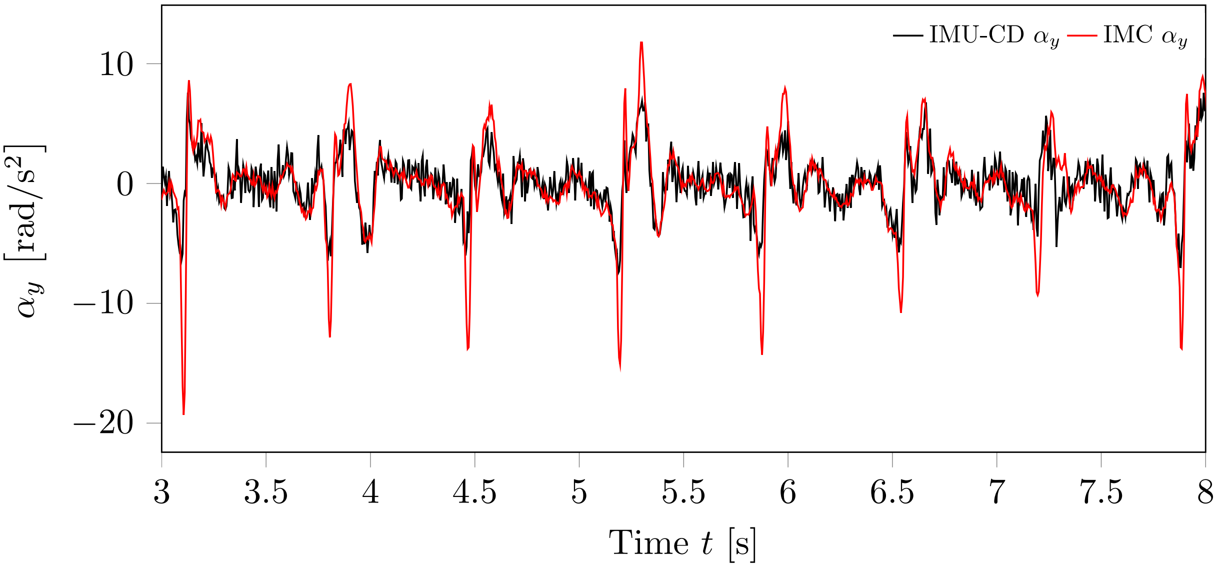

Fig. S1 Raw data of the trunk's angular acceleration around the transverse axis α_y for slow walking ($\sim 0.7\,\mathrm{m/s}$). The red curve represents the measurements by the inertial measurement cluster (IMC), the black curve the indirect measurement of the inertial measurement unit (IMU) using the central difference quotient (CD). The specific local extrema are resolved with notably lower amplitudes by numerical differentiation. This highlights the potential negative impact of numerical differentiation methods, particularly the risk of ignoring relevant motion characteristics.

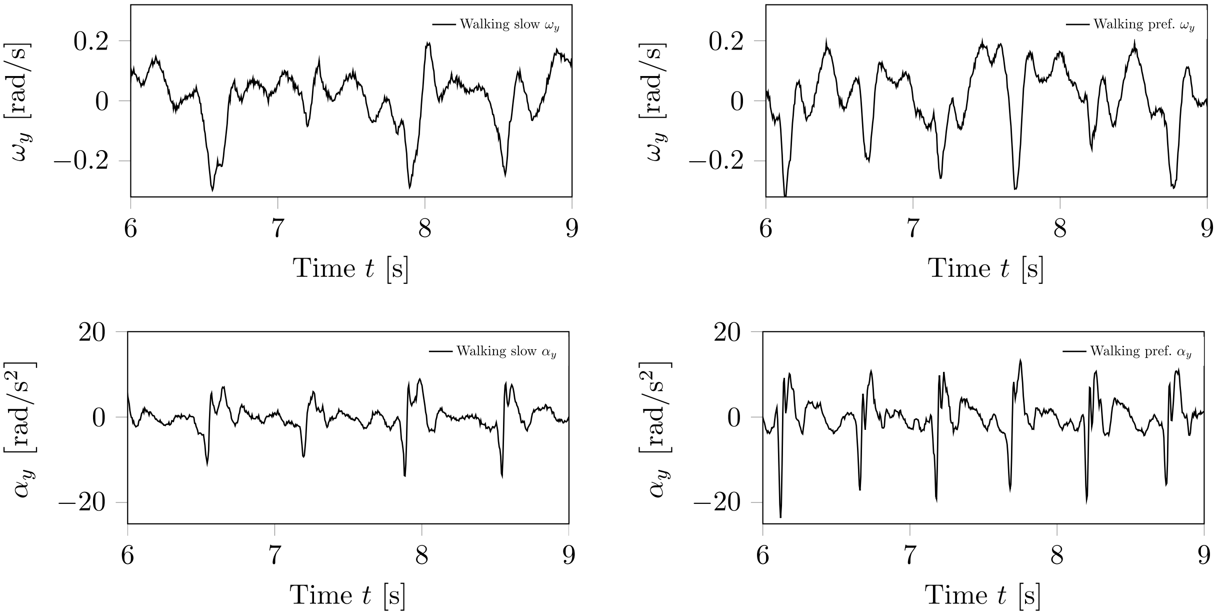

Fig. S2 Raw data of the trunk's angular velocity ω_y and acceleration α_y around the transverse axis for slow ((a) and (b)); $\approx 0.7\,\mathrm{m/s}$) and preferred walking ((c) and (d); $\approx 1.4\,\mathrm{m/s}$) evaluated by the inertial measurement cluster. For all panels the y-axis ticks are identical. The amplitude ranges of ω_y are quite similar for slow and preferred walking, whereas amplitude ranges of α_y diverge. This highlights the robustness of using α_y to distinguish more accurately between different types of gait velocities.

Supplementary Note 4

As outlined in [1], one essential requirement for the direct measurement principle was the linear independence of the relative vectors $\boldsymbol{r}_{i0}$ for $i = 1, 2, 3$, which then form a basis $\mathcal{G} = \{\boldsymbol{g}_1, \boldsymbol{g}_2, \boldsymbol{g}_3\}$ with $\boldsymbol{g}_1 = \boldsymbol{r}_{10}$, $\boldsymbol{g}_2 = \boldsymbol{r}_{20}$, and $\boldsymbol{g}_3 = \boldsymbol{r}_{30}$. Consequently, there is an existing and unique dual basis $\mathcal{G}' = \{\boldsymbol{g}^1, \boldsymbol{g}^2, \boldsymbol{g}^3\}$, defined by $\boldsymbol{g}_i \cdot \boldsymbol{g}^j = \delta_i^j$ (cf. [2]). Upon this, we exploit the linear independence of the $\boldsymbol{g}_i$ and their respective covectors $\boldsymbol{g}^i$ to unambiguously solve the well-known rigid body equation

$$\boldsymbol{a}_i = \boldsymbol{a}_0 + \dot{\boldsymbol{\omega}} \times \boldsymbol{r}_{i0} + \boldsymbol{\omega} \times (\boldsymbol{\omega} \times \boldsymbol{r}_{i0}), \qquad (1)$$

for $\dot{\boldsymbol{\omega}}$ leading to

$$\boldsymbol{g}^k \cdot \dot{\boldsymbol{\omega}} = \frac{1}{g}(\boldsymbol{a}_i - \boldsymbol{a}_0 - \boldsymbol{\omega} \times (\boldsymbol{\omega} \times \boldsymbol{r}_{i0})) \cdot \boldsymbol{r}_{j0}, \qquad (2)$$

as shown in [1, Eqn. (1)-(7)]. By utilizing the common calculation $\boldsymbol{g}^k = \frac{1}{g}\boldsymbol{g}_i \times \boldsymbol{g}_j$, where $g = (\boldsymbol{g}_1 \times \boldsymbol{g}_2) \cdot \boldsymbol{g}_3$ applies and, by interpreting the vectors as their coordinate matrices referenced in the IMC's non-inertial and orthogonal measurement coordinate frame, the coordinates α_k for $k = x, y, z$ of $\dot{\boldsymbol{\omega}} = \alpha_k \, \boldsymbol{e}_k$ can be calculated with

$$\begin{pmatrix} \alpha_x \\ \alpha_y \\ \alpha_z \end{pmatrix} = \begin{pmatrix} (\boldsymbol{r}_{10} \times \boldsymbol{r}_{20})^T \\ (\boldsymbol{r}_{20} \times \boldsymbol{r}_{30})^T \\ (\boldsymbol{r}_{30} \times \boldsymbol{r}_{10})^T \end{pmatrix}^{-1} \begin{pmatrix} (\boldsymbol{a}_1 - \boldsymbol{a}_0 - \boldsymbol{\omega} \times (\boldsymbol{\omega} \times \boldsymbol{r}_{10}))^T \, \boldsymbol{r}_{20} \\ (\boldsymbol{a}_2 - \boldsymbol{a}_0 - \boldsymbol{\omega} \times (\boldsymbol{\omega} \times \boldsymbol{r}_{20}))^T \, \boldsymbol{r}_{30} \\ (\boldsymbol{a}_3 - \boldsymbol{a}_0 - \boldsymbol{\omega} \times (\boldsymbol{\omega} \times \boldsymbol{r}_{30}))^T \, \boldsymbol{r}_{10} \end{pmatrix}, \qquad (3)$$

where $\boldsymbol{\omega}$ was extracted as the mean value of $\boldsymbol{\omega}_i$ for $i = 0, 1, 2, 3$. Note that the inverse matrix in Eqn. (3) always exists due to the linear independency of the vectors $\boldsymbol{r}_{i,0}$. Here, $\boldsymbol{e}_k$ are the basis vectors of IMC's non-inertial and orthogonal measurement coordinate frame.

Supplementary Note 5

In the event of a trip-like perturbation during forward walking, an external perturbation occurs during the swing phase of the swing leg. This perturbation delays the heel strike of the swinging leg, preventing the angular velocity of the non-actuated degree of freedom from being compensated by the heel strike. As a result of the balance recovery response to a trip-like perturbation, the trunk is accelerated rotationally around the transverse axis (Fig. S3 (b)). This angular acceleration of the trunk induces a moment of inertia related to the tipping edge, resulting in an opposite change in the total body angular momentum, thus reducing the angular velocity of the non-actuated degree of freedom. In the event of a slip-like perturbation, the foot begins to slide due to insufficient friction forces at the heel strike of the swinging leg. This causes a non-actuated degree of freedom around the tipping edge of the supporting foot. The body rotates posteriorly around the tipping edge (Fig. S3 (c)). To counteract this movement, the trunk is accelerated backwards, creating an inertia moment that is related to the tipping edge. This induces a change in the overall body angular momentum, which also reduces the angular velocity of the non-actuated degree of freedom. A similar principle for balance recovery responses exists even during quiet bipedal stance if an impulsive force acts on the person over a short but finite period. If the external force is large enough to create a non-actuated degree of freedom in the anteroposterior direction, a typical balance recovery response is that the trunk is accelerated in the respective direction, causing an inertia moment related to the tipping edge to counteract the non-actuated degree of freedom. Based on the evaluation coordinate system defined in Sec. Distinguishment between type of perturbations, the framework distinguishes between types of perturbations by analysing the trajectory of the trunk's angular velocity component along the transverse axis during a specific time interval that starts immediately after the perturbation onset. This interval is limited to half the duration of the perturbation, with a maximum of 0.25 s. During this period, we analysed the angular velocity signal to identify and compare the magnitudes of the first two local extrema that occurred immediately after the initial zero crossing of the measurement signal. We selected the extreme with the greater magnitude as the indicator. It is important to note that trip- or slip-like perturbations typically occur during walking and running. If the selected extreme has a positive sign with respect to the transverse axis, and the participant was standing before, it was classified as a loss of balance in the anterior direction during quiet bipedal stance (LOB-A). If the subject was in motion before the onset, it was classified as a trip-like perturbation. Similarly, if a negative sign of the extreme with respect to the transverse

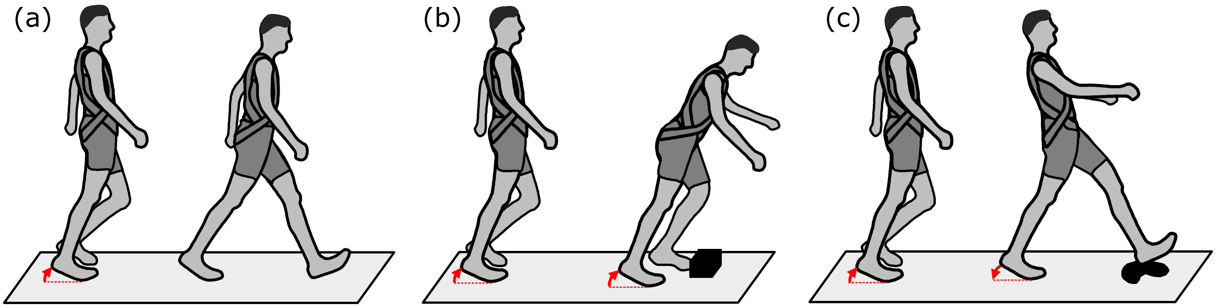

Fig. S3 Schematic illustration of unperturbed (a) and perturbed locomotion (b-c). In panel (a), the phase in-cluding a non-actuated degree of freedom (DOF, red arrow) and the end of this phase (heel strike) is shown for unperturbed locomotion. The event of a trip or slip (b) and (c) during forward walking may delay the heel strike to decelerate the angular velocity of the non-actuated degree of freedom, leading to a balance perturbation.

axis occurred as a consequence to the onset of the perturbation while standing, it was classified as a loss of balance in the posterior direction during quiet standing (LOB-P). If the participant was in motion prior to the perturbation onset, it was classified as a slip-like perturbation.

Supplementary Note 6

For each participant, the total measurement duration was limited to one hour to prevent potential fatigue and its impact on performance. Measurements were conducted at two different locations: one for participants P01 - P10 and another for participants P11 - P18. Posterior perturbations for loss of balance were performed later due to modifications in the perturbation system. Additionally, because of reported discomfort during posterior perturbations, only a subset of participants underwent these tests. For participants P15 - P18, activities of daily living could not be measured due to delays mainly caused by the required extensive explanation during testing.

The participant-specific sensitivity was 100 %, as no false negatives occurred. However, specificity varied among participants due to seven false positive detections. For participant P07, the participant-specific specificity was 90.9 %; for P02, it was 93.9 %; and for P08 and P09, it was 96.9 %.

Table S3 Summary of perturbed measurement trials (trip, slip, and loss of balance (LOB)) per participant.

Participant	Trip (cable pull)	Trip (element)	Slip (element)	LOB anterior	LOB posterior
P01	8	0	0	6	8
P02	7	0	0	6	6
P03	4	0	0	6	3
P04	6	0	0	6	6
P05	5	0	0	6	4
P06	7	0	0	6	0
P07	6	0	0	6	0
P08	5	0	0	6	0
P09	7	0	0	6	0
P10	7	0	0	4	0
P11	0	2	2	0	0
P12	0	2	2	0	0
P13	0	2	2	0	0
P14	0	2	2	0	0
P15	0	2	2	0	0
P16	0	2	2	0	0
P17	0	2	2	0	0
P18	0	2	2	0	0

Table S4 Summary of activities in daily living per participant. The entry per activity in daily living and per participant is divided in two numbers. The first number represents the number of true negative detected measurement trials. The second number represents the total measured trials of the respective activity in daily living. For participants P15 - P18 the activities in daily living could not be measured.

Participant	Pick and drop	Staircase	Stand up - Sit down	Walking	Running
P01	3 / 3	6 / 6	6 / 6	12 / 12	6 / 6
P02	3 / 3	6 / 6	4 / 6	12 / 12	6 / 6
P03	3 / 3	6 / 6	6 / 6	12 / 12	6 / 6
P04	3 / 3	6 / 6	6 / 6	12 / 12	6 / 6
P05	3 / 3	6 / 6	6 / 6	12 / 12	6 / 6
P06	3 / 3	6 / 6	6 / 6	12 / 12	6 / 6
P07	3 / 3	6 / 6	6 / 6	12 / 12	3 / 6
P08	3 / 3	6 / 6	6 / 6	12 / 12	5 / 6
P09	3 / 3	5 / 6	6 / 6	12 / 12	6 / 6
P10	3 / 3	6 / 6	6 / 6	12 / 12	6 / 6
P11	3 / 3	6 / 6	6 / 6	12 / 12	3 / 3
P12	3 / 3	6 / 6	6 / 6	12 / 12	3 / 3
P13	3 / 3	6 / 6	6 / 6	12 / 12	3 / 3
P14	3 / 3	4 / 4	6 / 6	12 / 12	3 / 3
P15	0	0	0	0	0
P16	0	0	0	0	0
P17	0	0	0	0	0
P18	0	0	0	0	0

References

[1] Gießler, M., Werth, J., Waltersberger, B., Karamanidis, K.: A wearable sensor and framework for accurate remote monitoring of human motion. Communications Engineering **3**(1), 20 (2024) https://doi.org/10.1038/s44172-024-00168-6

[2] Itskov, M.: Tensor Algebra and Tensor Analysis for Engineers: With Applications to Continuum Mechanics. Mathematical Engineering. Springer, Cham (2019). https://doi.org/10.1007/978-3-319-98806-1 . http://link.springer.com/10.1007/978-3-319-98806-1
