# Supplementary material for: A framework to automatically detect near-falls using a wearable inertial measurement cluster
Source: Commun Eng. 2024 Dec 16;3:181. doi: 10.1038/s44172-024-00325-x (PMC11649693; doi:10.1038/s44172-024-00325-x)
Supplement: Supplementary file 2 — nr-reporting-summary [file 44172_2024_325_MOESM2_ESM.pdf]

nature portfolio

Corresponding author(s): Maximilian Gießler

Last updated by author(s): Nov 5, 2024

Reporting Summary

Nature Portfolio wishes to improve the reproducibility of the work that we publish. This form provides structure for consistency and transparency in reporting. For further information on Nature Portfolio policies, see our Editorial Policies and the Editorial Policy Checklist.

Statistics

For all statistical analyses, confirm that the following items are present in the figure legend, table legend, main text, or Methods section.

n/a	Confirmed	
☐	☒	The exact sample size (*n*) for each experimental group/condition, given as a discrete number and unit of measurement
☐	☒	A statement on whether measurements were taken from distinct samples or whether the same sample was measured repeatedly
☒	☐	The statistical test(s) used AND whether they are one- or two-sided<br>*Only common tests should be described solely by name; describe more complex techniques in the Methods section.*
☒	☐	A description of all covariates tested
☒	☐	A description of any assumptions or corrections, such as tests of normality and adjustment for multiple comparisons
☒	☐	A full description of the statistical parameters including central tendency (e.g. means) or other basic estimates (e.g. regression coefficient) AND variation (e.g. standard deviation) or associated estimates of uncertainty (e.g. confidence intervals)
☒	☐	For null hypothesis testing, the test statistic (e.g. *F*, *t*, *r*) with confidence intervals, effect sizes, degrees of freedom and *P* value noted<br>*Give P values as exact values whenever suitable.*
☒	☐	For Bayesian analysis, information on the choice of priors and Markov chain Monte Carlo settings
☒	☐	For hierarchical and complex designs, identification of the appropriate level for tests and full reporting of outcomes
☒	☐	Estimates of effect sizes (e.g. Cohen's *d*, Pearson's *r*), indicating how they were calculated

Our web collection on statistics for biologists contains articles on many of the points above.

Software and code

Policy information about availability of computer code

Data collection	Regarding the real-world application, we used a custom code developed in C++ for the sensor. To collect the data, we used a custom code developed in the framework "Robot operating system 2 - Galactic" to store the data.
Data analysis	All analyses were performed using MATLAB (2023b, MathWorks, Natick, MA, USA).

For manuscripts utilizing custom algorithms or software that are central to the research but not yet described in published literature, software must be made available to editors and reviewers. We strongly encourage code deposition in a community repository (e.g. GitHub). See the Nature Portfolio guidelines for submitting code & software for further information.

Data

Policy information about availability of data

All manuscripts must include a data availability statement. This statement should provide the following information, where applicable:

- Accession codes, unique identifiers, or web links for publicly available datasets
- A description of any restrictions on data availability
- For clinical datasets or third party data, please ensure that the statement adheres to our policy

Datasets generated during and/or analysed during the current study are available on Figshare with identifier: https://doi.org/10.6084/m9.figshare.25569873

Human research participants

Policy information about studies involving human research participants and Sex and Gender in Research.

Reporting on sex and gender	The current study did not aim to investigate potential effects of either sex or gender on balance recovery. We intended to generally demonstrate whether our technology can assess human motion. Thus, no respective analyses were performed. As we inspected statistical assumption for each variable of interest (often including pooled data of 10 male and 8 female participants; self-reported prior to participation), we are confident that we have accounted for any potential statistical bias caused by sex or gender.
Population characteristics	As described in the methods section of the submitted version, our participants were healthy (i.e. neurological or musculoskeletal injuries or impairments limiting locomotion) and moderately active. The data is presented as averages and standard deviations for eight females and ten males:<br>Age: Females are on average 26 ± 4 years old and males are 31 ± 8 years old.<br>Height: Females on average 1.65 ± 0.06 meters and males 1.79 ± 0.10 meters.<br>Body Mass: Females on average 66.3 ± 7.7 kilograms and males 80.6 ± 10.4 kilograms.<br>BMI: Females had an average BMI of 24.4 ± 2.8 and males an average BMI of 25.2 ± 3.0.
Recruitment	Participants were recruited based on interest received upon information. We are confident that there were no bias caused by the recruitment procedure.
Ethics oversight	London South Bank University; School of Applied Science.

Note that full information on the approval of the study protocol must also be provided in the manuscript.

Field-specific reporting

Please select the one below that is the best fit for your research. If you are not sure, read the appropriate sections before making your selection.

☒ Life sciences ☐ Behavioural & social sciences ☐ Ecological, evolutionary & environmental sciences

For a reference copy of the document with all sections, see nature.com/documents/nr-reporting-summary-flat.pdf

Life sciences study design

All studies must disclose on these points even when the disclosure is negative.

Sample size	The current sample size was chosen based on our previous studies and respective effect sizes (e.g. Werth et al. 2022, Sci Rep 12) related to adaptation phenomena on balance control due to repeated perturbations; e.g. partial eta squared with n = 10 ranging from 0.536 to 0.775, depending on the variable of stability performance analysed).
Data exclusions	N/A
Replication	To assess the reproducibility of the sensor-framework system, we performed multiple trials for all tasks and analyzed trial-by-trial. Next, we previously performed several investigations on all five mechanical perturbation mechanisms and revealed consistent effects across studies whilst using different population groups and study designs. Thus, we are confident that all five tasks are reproducible.
Randomization	All participants underwent the same experimental protocol hence randomization was not relevant for the current study.
Blinding	We only assessed one group hence blinding was not relevant for the current study.

Reporting for specific materials, systems and methods

We require information from authors about some types of materials, experimental systems and methods used in many studies. Here, indicate whether each material, system or method listed is relevant to your study. If you are not sure if a list item applies to your research, read the appropriate section before selecting a response.

Materials & experimental systems

n/a	Involved in the study
☒	☐ Antibodies
☒	☐ Eukaryotic cell lines
☒	☐ Palaeontology and archaeology
☒	☐ Animals and other organisms
☒	☐ Clinical data
☒	☐ Dual use research of concern

Methods

n/a	Involved in the study
☒	☐ ChIP-seq
☒	☐ Flow cytometry
☒	☐ MRI-based neuroimaging
